# 30th Annual GP_2_A Medicinal Chemistry Conference

**DOI:** 10.3390/ph16030432

**Published:** 2023-03-12

**Authors:** Niamh M. O’Boyle, Jean-Jacques Helesbeux, Mary J. Meegan, Astrid Sasse, Elizabeth O’Shaughnessy, Alina Qaisar, Aoife Clancy, Florence McCarthy, Pascal Marchand

**Affiliations:** 1 School of Pharmacy and Pharmaceutical Sciences, Panoz Institute and Trinity Biomedical Sciences Institute, Trinity College Dublin, D02 PN40 Dublin, Ireland; 2 Department of SONAS, Université Angers, SONAS, SFR 4207 QUASAV, F-49000 Angers, France; 3 School of Chemistry and ABCRF, University College Cork, T12 K8AF Cork, Ireland; 4 Cibles et Médicaments des Infections et de l’Immunité, IICiMed, Nantes Université, UR 1155, F-44000 Nantes, France

**Keywords:** medicinal chemistry, drug design, chemical biology, chemical tools, ligand kinetics, pharmaceutical chemistry

## Abstract

The Group for the Promotion of Pharmaceutical Chemistry in Academia (GP_2_A) held their 30th annual conference in August 2022 in Trinity College Dublin, Ireland. There were 9 keynote presentations, 10 early career researcher presentations and 41 poster presentations.

## 1. Introduction

The Group for the Promotion of Pharmaceutical chemistry in Academia (GP_2_A) celebrated the milestone of 30 years of networking and sharing ideas in 2022. The group was previously known as the Groupement des Pharmacochimistes de l’Arc Atlantique but has expanded from the initial members from the ‘Atlantic Arc’. GP_2_A hosted their 30th annual conference in Dublin, Ireland from 24th to 26th August. This annual event brings together participants from across Europe and further afield, with the aim of connecting university and industrial researchers working in the field of medicinal chemistry and chemical biology. This congress is known for its high scientific standing, its friendly atmosphere and the intensity of the exchanges of experience that it allows between senior and young researchers (doctoral or post-doctoral students). The conference was held in the Hamilton building in Trinity College Dublin, hosted by the Trinity School of Pharmacy and Pharmaceutical Sciences. 

Over a hundred researchers from France, Ireland, Germany, United Kingdom, Spain, Portugal, Belgium, Ukraine, Brazil, Australia and United Arab Emirates participated in this congress. Travel support was provided to Ukrainian scientists to attend the event. Nine internationally renowned speakers shared their research in plenary lectures (keynote lectures 2.1–2.9, below). We welcomed Prof. Isabel Rozas (Trinity College Dublin), Dr. James Hodgkinson (University of Leicester), Prof. Dr. Anna K. H. Hirsch (Helmholtz Institute for Pharmaceutical Research Saarland), Dr. Andrew Beekman (University of East Anglia), Jun.-Prof. Dr. Matthias Gehringer (University of Tübingen), Dr. Kurt Hoogewijs (NUI Galway), Prof. Christa Elisabeth Müller (University of Bonn), Prof. Swen Hoelder (Institute of Cancer Research, UK) and Prof. Rebecca Deprez-Poulain (University of Lille) to speak at the conference. 

There were a further ten presentations from early career researchers from Europe and Australia, on topics from aza-heterocyclic structures in medicinal chemistry to pan-PPAR agonists to RSV small molecule entry inhibitors and more (early career researcher presentations 3.1–3.10, below). Researchers also presented 41 posters on diverse topics (poster presentations 4.1–4.41, below). Nine prizes were awarded and the prizewinners were:

- Best Presentation by a PhD Student: Magdalena Wojciechowski, University of Münster, Institute of Pharmaceutical and Medicinal Chemistry. Prize sponsored by the European Journal of Medicinal Chemistry.
- Runner-Up Presentation by a PhD Student: Christina Kosch, Helmholtz Institute of Pharmaceutical Research Saarland (HIPS). Prize sponsored by RSC Medicinal Chemistry.
- Best Presentation by a Postdoctoral Researcher: Dr. Manuela Jörg, Medicinal Chemistry, Monash University. Prize sponsored by Almac Group.
- Runner-Up Presentation by a Postdoctoral Researcher: Dr. Mariia Nesterkina, Helmholtz Institute of Pharmaceutical Research Saarland (HIPS). Prize sponsored by Pharmaceuticals.
- Best Poster Presentations: Julian Breidenbach (Rheinische Friedrich-Wilhelms-Universität Bonn), Hugo Bloux (CERMN, University of Caen), Aline Renata Pavan (UNESP, Brazil), Rhys Francis (The University of Nottingham), Nikolina Stipaničev (Trinity College Dublin). Prizes sponsored by the European Journal of Medicinal Chemistry, European Journal of Medicinal Chemistry, RSC Medicinal Chemistry, ThermoFisher, and CEM.

There was a nice social side to the conference, with a welcome reception on the first evening in the Dining Hall of Trinity College Dublin. This included a popular ‘photo booth’ which provided great amusement to the delegates. Conference delegates could also participate in a walking tour of Dublin. A highlight was the conference banquet dinner and Irish music and dance show in the Abbey Tavern, Howth. 

## 2. Keynote Lectures

### 2.1. Exploring Heterobifunctional Molecules and Class-I Histone Deacetylase Enzymes in Complexes

HodgkinsonJames T.SchwabeJohn W.R.CowleyShaun M.SmalleyJoshua P.BakerIndia M.BowmanKaren J.Leicester Institute of Structural and Chemical Biology, School of Chemistry, University of Leicester, University Road, Leicester LE1 7RH, UK; JTHodgkinson@le.ac.uk

**Figure 1:**
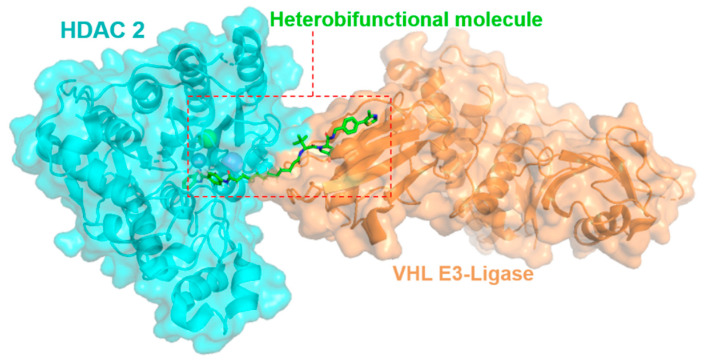
Model of HDAC2 and the Von Hippel-Lindau E3-ligase brought into proximity with a heterobifunctional molecule.

Histone Deacetylase enzymes (HDACs) have proved to be viable drug targets in hematologic cancers and offer promise in neurodegenerative disorders and cardiovascular diseases [1]. Of the eleven Zn^2+^ dependent HDAC enzymes in the human body, HDAC 1, 2 & 3 are uniquely localised in the nucleus of the cell and exist in several large multi-protein co-repressor complexes regulating chromatin structure and gene transcription [2,3]. Current drugs targeting HDACs in the clinic exhibit limited selectivity for the eleven HDAC isoforms and also lack selectivity for the multi-protein complexes in which HDAC 1, 2 and 3 exist in vivo. I will describe our efforts in the design and biological evaluation of Class I HDAC 1, 2 & 3 heterobifunctional molecules to study the induced proteasome mediated degradation of these important enzymes in cells (Figure 1) [4,5,6].

### 2.2. Targeting the Protein Kinases’ Cysteinome: Design of Covalent MPS1/TTK and BMX Inhibitors

GehringerMatthias^1^,^2^1Department of Pharmaceutical & Medicinal Chemistry, Institute of Pharmaceutical Sciences, Eberhard Karls University Tübingen, Auf der Morgenstelle 8, 72076 Tübingen, Germany; matthias.gehringer@uni-tuebingen.de2Cluster of Excellence iFIT (EXC 2180) ‘Image-Guided & Functionally Instructed Tumor Therapies’, University of Tübingen, 72076 Tübingen, Germany

Protein kinases are among the major drug targets of the 21^st^ century with over 70 inhibitors approved since Imatinib, the first small molecule protein kinase inhibitor, entered the market in 2001. Despite the great success of protein kinase inhibitors, achieving selectivity for individual kinases remains an obstacle due to the highly conserved structure of the >500 protein kinases making up the human “kinome” [7]. A powerful approach for generating highly selective kinase inhibitors relies on covalent targeting of poorly conserved, non-catalytic cysteines, which are found at various locations in or around the ATP binding site of approx. 200 protein kinases [8]. This approach has successfully been employed in chemical probe development and drug discovery, as highlighted by currently eight FDA-approved covalent protein kinase inhibitors and many more in clinical trials [9]. In my talk, I will present two recent case studies highlighting the successful design and synthesis of covalent inhibitors targeting the protein kinases MPS1 (monopolar spindle kinase 1, also called TTK) and BMX (bone marrow tyrosine kinase on chromosome X). 

MPS1/TTK is a key regulator of the spindle assembly checkpoint orchestrating chromosome segregation and considered a promising target for the treatment of different cancers including triple negative breast cancer and colorectal carcinoma. Notably, several reversible MPS1 inhibitors have entered clinical trials. Following a structure-based design approach, we were able to address a poorly conserved cysteine in the so-called hinge-region of the kinase to generate the first covalent MPS1 inhibitor, RMS07 [10]. BMX is a non-receptor tyrosine kinase belonging to the TEC-family and a close relative to Bruton’s tyrosine kinase, which is the target of FDA-approved drugs such as Ibrutinib and Acalabrutinib. While the biology of BTK is well-understood, much less is known about the physiological role of BMX and no specific BMX inhibitors are currently available to be used as chemical probes. Using a covalent inhibitor from our Janus kinase 3 (JAK3) program, which showed off-target activity on BMX, as a starting point, we employed structure-based design to generate low nanomolar dual BMX/BTK inhibitors with high selectivity against JAK3 and other kinases with an equivalent cysteine [11]. In a recent second round of optimization, selectivity against BTK could be improved by targeting subtle structural differences between these two kinases.

### 2.3. A Tricycle Journey to In Vivo Degraders of the Oncogenic Transcription Factor BCL6

HoelderSwenThe Institute of Cancer Research, 123 Old Brompton Road, London SW7 3RP, UK; swen.hoelder@icr.ac.uk

BCL6 is an oncogenic, transcriptional repressor critical for antibody maturation in B cells. Expression of BCL6 is upregulated through recurrent gene changes in a large fraction of lymphoma patients making BCL6 an attractive target. In the presentation, I will summarise our discovery of BCL6 degraders suitable for in vivo use. We started from weakly potent HTS hits that we developed in into initial degraders [12]. These early degraders suffered from high lipophilicity and modest activity. Our BCL6 degraders consisted of two key elements: (1) a central benzimidazolone core that bound to BCL6 and (2) a substituted piperidine that conveys the ability to induce BCL6 degradation. To identify advanced degraders, we discovered a tricyclic core that showed hundredfold tighter binding to BCL6 than our initial benzimidazolone core [13]. In addition, we identified alternative piperidine moieties that we significantly less hydrophobic but still capable to induced degradation [14]. Combining both elements led to BCL6 degrader CCT373566. CCT373566 is an exquisitely potent degrader of BCL6 and showed an overall favourable profile including satisfactory PK properties. BID dosing of CCT373566 allows near complete and sustained depletion of BCL6 in mouse models.

### 2.4. IDE and ERAP Inhibitors Discovered by KTGS and Their Use in Therapeutics

Deprez-PoulainRebeccaInserm, Institut Pasteur de Lille, U1177 Drugs & Molecules for Living Systems, Universite de Lille, F-59000 Lille, France; rebecca.deprez@univ-lille.fr

Kinetic Target-Guided Synthesis (KTGS) is an elegant and efficacious protein-templated synthesis that uses the protein of interest to catalyze the synthesis of its own ligands. In KTGS specifically, the biological target accelerates an irreversible reaction between a pair of reagents by stabilizing a productive configuration of the ternary complex. The in situ “click” chemistry is the most widely used KTGS reaction.

After describing the successes and challenges of KTGS for drug discovery, we will exemplify its use for the discovery of metalloprotease inhibitors. 

We disclose first how we identify insulin degrading enzyme (IDE) inhibitors. These are used to explore the role of this enzyme in liver and are leads for therapeutic application in oncology. At last, we disclose some of our most recent work on metalloproteases of the M1 family, that led to the discovery of the first nanomolar selective inhibitors of the aminopeptidase of the endoplasmic reticulum ERAP2, for modulation of the antigenic presentation in immune diseases and in immune-oncology.

### 2.5. Structure-Affinity Relationship Study of a Potential Therapeutic Peptide for the Treatment of MELAS

HoogewijsKurtSchool of Biological and Chemical Sciences, University of Galway, University Road, Galway H91 TK33, Ireland

Mitochondrial diseases are caused by mutations in the mtDNA. The most common point mutation m.3243A>G affects the structure and function of mitochondrial tRNALeu(UUR) and causes the disease MELAS. There are currently no treatments for MELAS, but a 16 amino acid peptide reported to be rescuing cellular defects is an excellent starting point. We are currently designing peptidomimetics with enhanced resistance to proteolysis in cells and mitochondria. We have synthesized over 100 analogues and screened them using MST. Current efforts are focussed on the design of 2nd generation compounds based on previously obtained structure-affinity relationship studies.

### 2.6. New Strategies for the Identification and Control of Disease Relevant Protein-Protein Interactions

BeekmanAndrewSchool of Pharmacy, University of East Anglia, Norwich Research Park, Norwich, Norfolk, NR4 7TJ, UK; A.Beekman@uea.ac.uk

Protein-protein interactions control all biological processes but are difficult to drug due to their large hydrophobic interaction surfaces. However, protein-interactions are commonly controlled by hotspot binding sites at the interaction surface [15]. Peptides derived from the natural protein partner are key for the development of protein interaction modulators, informing the design of small molecules. Peptides are essential chemical probes, but often suffer from poor stability and cellular uptake, making small molecules more desirable for drug development.

**Figure 2:**
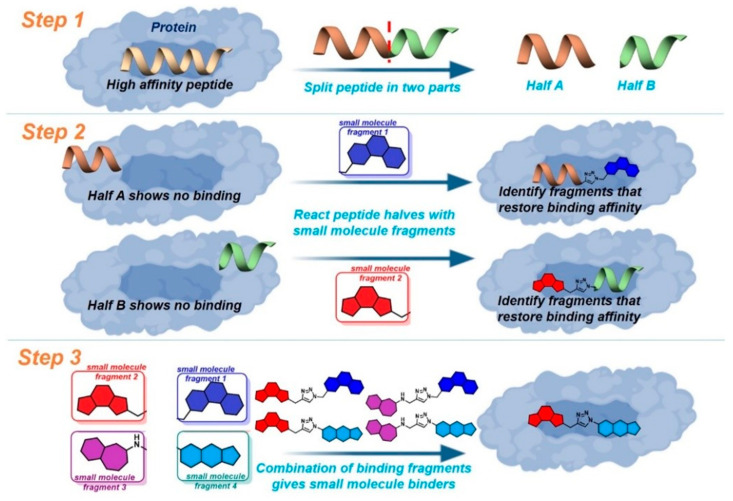
Schematic of peptide-directed binding.

In 2017, we disclosed a highly efficient approach to inhibiting protein-protein interactions [16]. Using a peptide as framework we designed small molecules by combining peptide design with fragment based chemistry (Figure 2). The outcome was very impressive–out of only 20 compounds, 50% had IC_50_ values below 100 µM and 25% below 1 µM for target binding.

This presentation will describe our advances on this technique and its application to new protein-protein interactions [17,18].

### 2.7. Addressing Unusual Anti-Infective Targets

HirschAnna K. H.Helmholtz Institute for Pharmaceutical Research Saarland (HIPS), 66123 Saarbrücken, Germany; anna.hirsch@helmholtz-hips.de

The challenges associated with anti-infective drug-discovery programmes can be tackled by combining several established and unprecedented hit-identification strategies with phenotypic antibacterial screening [19]. I will illustrate this approach with a selection of un(der)explored targets. The first is a vitamin transporter from the energy-coupling factor (ECF) class, which is unique to Gram-positive bacteria [20]. Here, we report on the structure-based virtual screening (SBVS), design, synthesis and structure–activity relationships of the first classes of selective, antibacterial inhibitors of the energy-coupling factor (ECF) transporters with good in vitro and whole-cell activity and a good in vitro ADMET and in vivo PK profiles [21]. A newly established cell-based uptake assay in *Lactobacillus casei* greatly facilitated our screening and hit-to-lead optimisation campaign [22]. 

The second is the β-subunit of the bacterial DNA polymerase III (sliding clamp, DnaN), an attractive antibacterial target. We pursued several hit-identification strategies [23], including a SBVS campaign, affording novel chemotypes with micromolar affinity and promising antibacterial activity. Mode-of-action studies confirmed DnaN as the molecular target. The new compound displays broad-spectrum antibacterial activity against mycobacteria, Gram-positive and Gram-negative pathogens also against multidrug-resistant bacteria with no cytotoxicity and good in vivo PK profiles. 

Finally, we succeeded in fragment merging and linking, affording highly selective and potent inhibitors of the extracellular metalloprotease and virulence factor of *Pseudomonas aeruginosa*, the elastase LasB [24,25]. Multiparameter optimisation is currently ongoing based on extensive in vitro and ex vivo profiling, including the establishment of complex biological assays. Our approach promises to deliver the urgently needed anti-infective agents featuring both new chemical scaffolds and unprecedented modes of action. Multiparameter optimisation is currently ongoing based on extensive in vitro, whole-cell, ex vivo and in vivo profiling, including the establishment of complex biological assays. A particular emphasis will be placed on the lead optimisation of frontrunners for permeation and achieving good lung exposure.

### 2.8. Targeting Membrane Proteins Involved in Purinergic Signalling–Important Players in Inflammation, Immunity and Cancer

MüllerChrista E.Department of Pharmaceutical & Medicinal Chemistry, University of Bonn, 53121 Bonn, Germany; christa.mueller@uni-bonn.de

**Figure 3:**
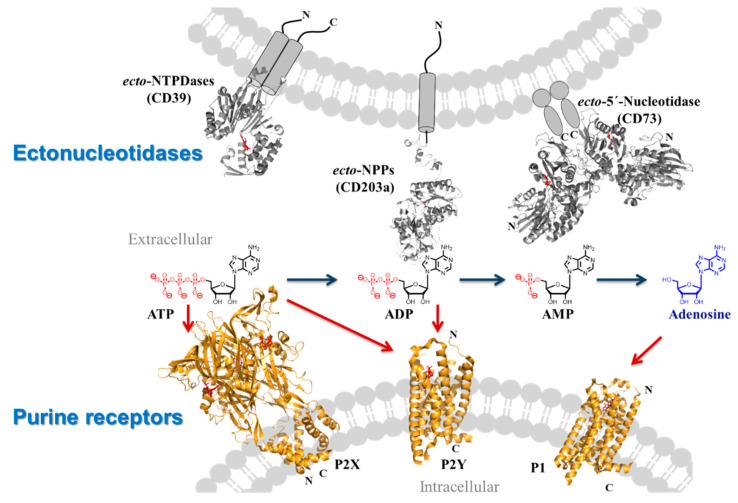
Activation pathway of purine P2Y and P2X receptors.

Extracellular adenosine triphosphate (ATP) acts as a pro-inflammatory danger signal via activation of purine P2Y and P2X receptors (Figure 3). In contrast, its corresponding nucleoside adenosine is a strongly immunosuppressive agent activating G protein-coupled P1 (adenosine) receptors.

Cancer tissues can release large amounts of the nucleotide ATP, which is immediately hydrolyzed by ectonucleotidases, upregulated on many cancer cells, leading to the production of adenosine. Activation of adenosine A_2A_ and A_2B_ receptors results in cancer-promoting, angiogenic, pro-metastatic, and strongly immunosuppressive effects.

The balance between the impact of pro-inflammatory ATP and anti-inflammatory adenosine can be modulated by ectonucleotidase inhibitors, or by activation or blockade of purine receptors. Recent progress of our laboratory in the identification and optimization of purine receptor antagonists and ectonucleotidase inhibitors by convergent approaches, utilizing structural biology, will be presented. These tool compounds, including labeled derivatives, are used to study their targets’ role in health and disease. Moreover, they have potential for further development as novel drugs.

### 2.9. Where Targeting DNA Can Take You…

RozasIsabelTrinity Biomedical Sciences Institute, School of Chemistry, Trinity College Dublin, The University of Dublin, 152-160 Pearse St., Dublin, Ireland, D02 R590; rozasi@tcd.ie

**Figure 4:**
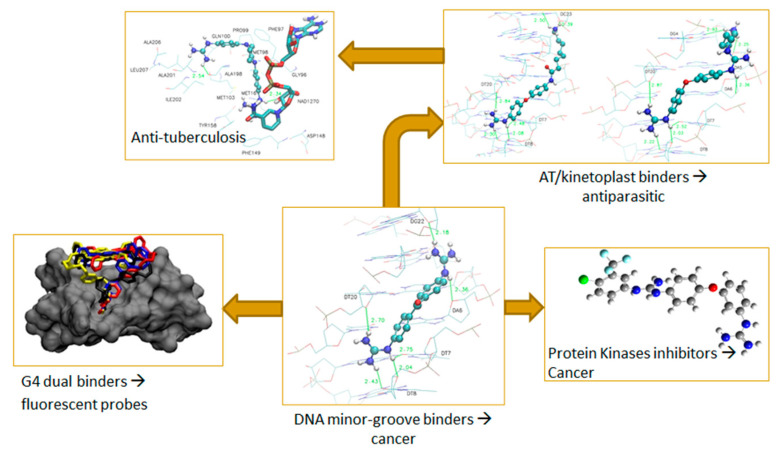
DNA minor-groove binders for different applications.

During more than 20 years our group has computationally designed, prepared, and biologically evaluated a large number of compounds targeting the minor groove of DNA [26,27,28,29]. In this talk, I will present the results that we obtained from the study of these DNA minor groove binders, how the results obtained informed the different modifications that we carried out, and how these studies opened research pathways towards cancer or antiparasitic agents as well as other targets such as protein kinases, or tuberculosis related enzymes (Figure 4). As part of this ‘journey’ I will end presenting some novel research on targeting guanine quadruplexes ligand with the aim to produce fluorescent probes [30]. 

## 3. Early Career Researcher Presentations

### 3.1. Identification and Structure-Activity Relationship Profiling of Positive Allosteric Modulators Targeting Muscarinic Acetylcholine Receptors

JörgManuela^1^,^2^van der WesthuizenEmma T.^3^ValantCeline^3^ThalDavid M.^3^CapuanoBen^1^ChristopoulosArthur^3^ScammellsPeter J.^1^1Medicinal Chemistry, Faculty of Pharmacy and Pharmaceutical Sciences, Monash University, Parkville 3052, Australia2School of Natural and Environmental Sciences, Newcastle University, Newcastle upon Tyne NE1 7RU, UK3Drug Discovery Biology, Monash University, Parkville 3052, Australia; manuela.jorg@monash.edu

Muscarinic acetylcholine receptors (mAChRs) have been recognized as promising drug targets, however, the design of selective orthosteric ligands has proven to be extremely challenging due to the highly conserved orthosteric site across all the muscarinic receptor subtypes (M1-M5 mAChRs). Thus, there has been growing interest in the identification and optimization of allosteric ligands targeting the less conserved regions of the mAChRs. 

Our group has successfully reported on the design and synthesis of several novel families of M1 mAChR positive allosteric modulators (PAMs) [31,32,33,34,35,36,37] and most recently M4 mAChR PAMs (unpublished data). We evaluated the analogues using innovative protocols allowing for higher throughput characterisation of PAMs, while also capturing their complex pharmacological profiles. This work resulted in the identification of mAChR PAMs with distinct profiles, which will further improve our ability to predict different biological outcomes by linking unique signalling profiles of mAChR PAMs with the manifestation of therapeutic and/or adverse effects in preclinical animal models. 

Ongoing research includes the synthesis of structurally novel M4 PAMs based on a scaffold-hoping approach as well as the use of cryogenic electron microscopy (cryo-EM) and DNA-encoded libraries (DELs) to further advance key analogues as well as identifying structurally novel families of mAChRs PAMs with improved affinity, selectivity and CNS permeability. 

### 3.2. New Aza-Heterocyclic Structures for Feeding the Drug Space in Medicinal Chemistry

LeblancJohann^1^AllainmatMonique Mathé^1^GrosseSandrine^2^GuillemontJérôme^3^LebretonJacques^1^TessierArnaud^1^1CNRS, Nantes Université, CEISAM, UMR 6230, 2 rue de la Houssinière, BP 92208, F-44000 Nantes, France; arnaud.tessier@univ-nantes.fr2Janssen Research & Development, Turnhoutseweg 30, 2340 Beerse, Belgium3NovAliX on-site Janssen-Cilag, Centre de recherche Pharma, Campus de Maigremont, 27106 Val de Reuil, France

**Figure 5:**
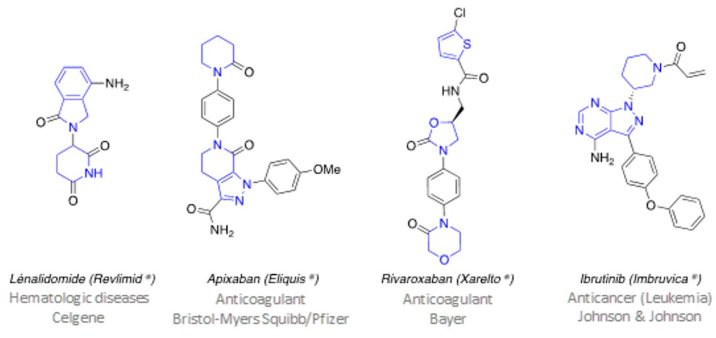
Recent big pharma’s blockbusters featuring key aza-heterocyclic compounds.

Omnipresent both in nature and in synthetic chemistry, heterocycles constitute essential molecular units that are found in natural products, in medicinal chemistry, in materials, in catalysis, etc. Their structural characteristics directly influence the physicochemical properties of the molecules that contain these heterocyclic units (conformation, solubility, electronic density, etc.), thus justifying their role and their attractiveness in each of these areas. Within this chemical space, nitrogenous heterocycles hold a primordial place, as for example in pharmaceutical chemistry, where nearly 60% of drugs approved by the FDA are composed of at least one nitrogenous heterocycle (Figure 5), with a clear predominance of piperidine, pyridine or piperazine units [38,39].

This communication will present the preparation of cyclic amidrazones as new non-aromatic aza-heterocyclic structures by original synthetic methodologies implemented within our research team. The synthetic developments of these aza-heterocycles aim to integrate new functionalities on original heterocyclic units, and to access new families of functionalized heterocycles. Given the strong predominance of nitrogen heterocycles in medicinal chemistry, the exploitation of these original aza-heterocyclic structures will contribute to pave the way of new bioisosteric scaffolds within the drug space of pharma blockbusters. 

The authors are very grateful to Janssen Pharmaceuticals for providing financial support to this research programme. 

### 3.3. A novel Polycerasoidol Analogue with Pan-PPAR Agonism, Anti-Inflammatory Effects and Amelioration of Metabolic Abnormalities in Ob/Ob Mice

Villarroel-VicenteCarlos^1^,^2^GarcíaAinhoa^2^MarquesPatrice^1^,^2^VilaLaura^1^HennuyerNathalie^3^StaelsBart^3^CortesDiego^1^,^2^SanzMaría Jesús^1^,^2^CabedoNuria^1^,^2^1Institute of Health Research-INCLIVA, Hospital Clínico Universitario de Valencia, 46010 Valencia, Spain2Pharmacology Department, Universidad de Valencia, 46100 Valencia, Spain; dcortes@uv.es; ncabedo@uv.es3Université de Lille, Inserm, CHU Lille, Institut Pasteur de Lille, U-1011-EGID, F-5900 Lille, France

**Figure 6:**
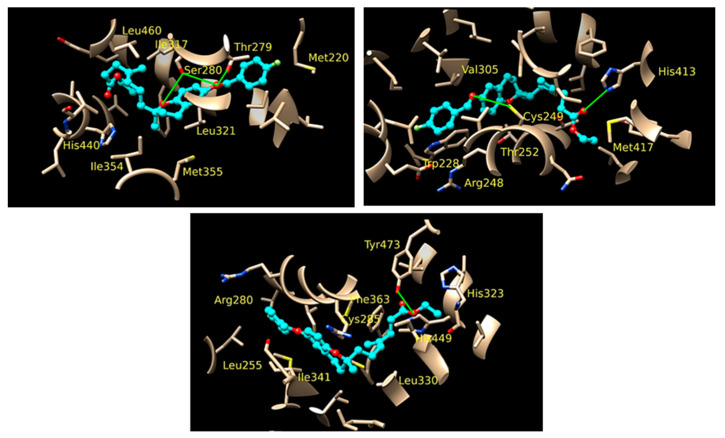
Molecular docking on PPARα,β,γ receptors of BP-2.

Peroxisome proliferator-activated receptors (PPARs) have been described as targets to manage metabolic diseases, including metabolic syndrome, type 2 diabetes, atherogenic dyslipemia and non-alcoholic fat liver disease (NAFLD). Given that selective and full PPARγ agonists have been related to severe adverse effects, pan-PPAR agonists have raised a special attention for therapeutic purposes. In an effort to discover a safer and more effective dual or pan-PPAR agonist, we designed a prenylated benzopyran (BP-2) through a Grignard reaction sequence, followed by Johnson-Claisen rearrangement and subsequent Wittig olefination. Molecular modelling studies identified those amino acid residues relevant for ligand binding to human PPAR receptors (Figure 6). To know its potential anti-inflammatory and metabolic effects, BP-2 was evaluated on TNFα-induced leukocyte adhesion and investigated after administration in obese mice model. Our results showed that compound BP-2 concentration-dependently reduced TNF**α**-induced endothelial mononuclear cell adhesion via RXRα/PPARγ interactions. BP-2 also down-regulated TNFα-induced endothelial adhesion molecules and inhibited p38MAPK and NF-κB activation. In addition, BP-2 was able to reduce inflammation in *ob/ob* mice and ameliorate lipid parameters [40]. Therefore, compound BP-2 can be considered as a novel lead candidate for the treatment of cardiometabolic disorders.

This work was funded by grants PFIS (FI19/00153) to C.V.V and Miguel Servet programme (CPII20/00010) to N.C. from the Carlos III Health Institute (ISCIII) co-funded by the European Social Fund (ESF) “Investing in your future”, and by grants SAF2017-89714-R, PID2020-120336RB-I00, PI18/01450, PI21/02045, AICO/2021/081, APOTIP/2020/011 and PROMETEO/2019/032 from the SMSI, GVA, ISCIII and co-funded by European Regional Development Fund (ERDF). 

### 3.4. Triagonist Peptide Designed to Activate Incretin/Glucagon System

VishnoiShubhamBhattacharyaShayonThompsonDamienSynthesis and Solid State Pharmaceutical Centre (SSPC), Department of Physics, Bernal Institute, University of Limerick, V94T9PX Limerick, Ireland; shubham.vishnoi@ul.ie

**Figure 7:**
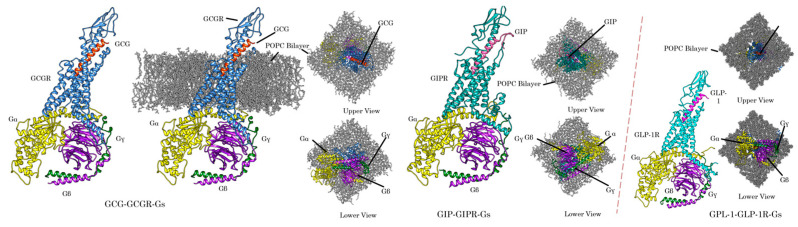
Computational design of endogenous agonist-bound GCG/GIP/GLP-1 receptors in complex with heterotrimeric Gs proteins, composed of Gα, Gβ, and Gγ subunits.

Obesity is the leading risk factor for prediabetes, diabetes, and the aggravation of pregestational (also called preexisting) diabetes by negatively affecting lipid metabolism, insulin sensitivity, and β-cell functioning. The sharp global rise in the prevalence of obesity and its associated comorbidities (T2DM, non-alcoholic steatohepatitis (NASH)/nonalcoholic fatty liver disease (NAFLD)) is rapidly driving the need to discover new pharmacotherapy to treat the metabolic disorders [41]. The study suggests that effective bodyweight loss not only prevents or delays the onset of T2DM but also enhances the therapeutic response to diabetes drugs including basal insulin in T2DM patients [42]. Several drugs for metabolic problems (diabetes and obesity) are based on the gut hormones GCG/GLP-1 that modulate the hepatic glycogen and fat content, and control appetite and blood glucose level. The proposed talk will be about our recent work that reports a detailed atomistic-level unimolecular GCG/GLP-1 receptor co-agonist that could be developed to provide effective bodyweight loss with T2DM management (Figure 7). Although GLP-1, GIP, and glucagon have some overlapping functionality, their combined use leads to synergistic effects on diabetes and related metabolic disease. So, this talk will also highlight exploring three class B1 receptor specificity and their potential for agonism with a designed tri-agonist by including the GIP receptor in the study along with the GCG/GLP-1 receptor. 

### 3.5. Design and Synthesis of Novel Drugs for the Treatment of Cryptosporidiosis

UpadhyayAmit^1^,^2^O’SullivanTimothy P.^1^,^2^,^3^1School of Chemistry, University College Cork, T12 K8AF Cork, Ireland; 119222561@umail.ucc.ie2Analytical and Biological Chemistry Research Facility, University College Cork, T12 K8AF Cork, Ireland3School of Pharmacy, University College Cork, T12 K8AF Cork, Ireland

**Figure 8:**
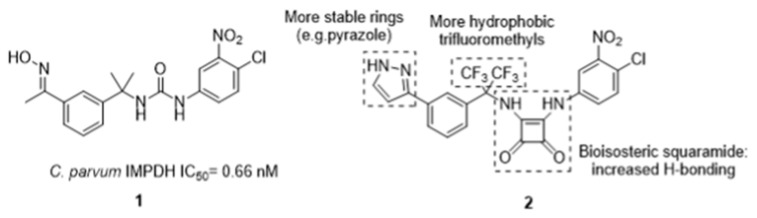
Comparison of lead structure with proposed changes.

Despite being one of the most widespread diarrheal diseases, there are no effective treatments available for cryptosporidiosis, which is caused by an oocyst-forming protozoan, *Cryptosporidium* [43]. Severe illness resulting from *Cryptosporidium* infection can be life-threatening, especially in young children and people with weak immunity such as patients with HIV [44]. Cryptosporidiosis outbreaks have been reported in countries all over the globe, including Ireland. Inosine monophosphate dehydrogenase (IMPDH) has gained the attention of researchers in search of novel treatments of cryptosporidiosis. In the protozoan, IMPDH is involved in the de novo synthesis of guanine nucleotides [45]. This enzyme has a bacterial origin and is notably distinct from host cell enzymes. Cuny et al.have identified urea 1 as a potent inhibitor of *Cp*IMPDH (Figure 8) [46]. However, 1 is prone to rapid in vivo metabolism. 

In this project, we have proposed several modifications to the lead structure. These include replacement of the existing urea with a bioisosteric squaramide. Squaramide 2 should display enhanced metabolic stability, increased duration of action and higher biological activity. The replacement of an unstable oxime with a chemically robust heteroaromatic ring is also envisaged. Herein, we present our work to date on the design and synthesis of novel *Cryptosporidium* IMPDH inhibitors incorporating these changes. We also compare the efficiency of two different preparative routes which incorporate key early- or late-stage Suzuki couplings respectively. 

We would like to acknowledge the Irish Research Council for financial support. 

### 3.6. Gold(I) Mediated Radio-Iododecarboxylation: Application in Nuclear Medicine

KhouyaAhmed Ait^1^BlouxHugo^1^DubostEmmanuelle^1^,^2^FabisFrédéric^1^CaillyThomas^1^,^2^,^3^,^4^1Centre d’Etudes et de Recherche sur le Medicament de Normandie, Normandie Université, 14000 Caen, France; ahmed.aitkhouya@unicaen.fr2IMOGERE, Normandie Université, 14000 Caen, France3Department of Nuclear Medicine, CHU Côte de Nacre, 14000 Caen, France4Institut Blood and Brain @ Caen Normandie (BB@C), 14000 Caen, France

**Figure 9:**
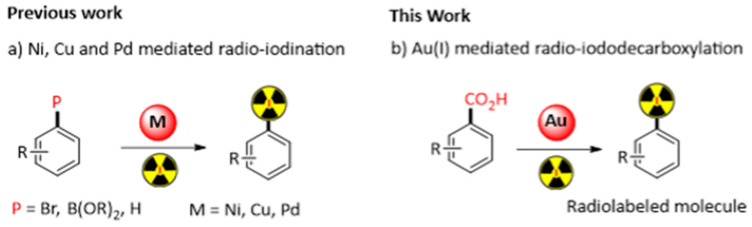
Transition metal mediated radio-iodination of arene.

Labelling of (bio)molecules with radioactive isotopes is of high interest to the scientific community, as it strongly impacts the discovery process in life sciences and nuclear medicine. Radio-labelled molecules have been used to access biochemical reactions by *(i)* measuring the in vivo distribution of a substance or *(ii)* performing in vitro binding assays or RadioImmunoAssay (RIA) [47]. In nuclear medicine, radio-therapeutics for RadioIsotope Therapy (RIT) [48] and radio-tracers for molecular imaging experiments such as Positron Emission Tomography (PET) or Single Photon Emission Computed Tomography (SPECT) have been described [49]. In this context, four iodine radioactive-isotopes can be used, each one with a specific application: ^123^I and ^124^I for SPECT and PET imaging respectively, ^125^I for binding studies, and ^131^I for radiotherapy [50]. Considering the difficulties and the cost of developing a radiolabeling process, the development of efficient synthetic methods is highly desirable. Aiming to overcome these issues, a variety of new transformations mediated by transition metal (Ni, Cu and Pd) have been developed in recent years (Figure 9a) [51]. Inspired by the gold(I)-medieted decarboxylation of arene described by Larrosa [52], our team recently demonstrated that a carboxylic acid function can be used to promote radio-iodination. In this study, we will present the straightforward decarboxylative gold(I) mediated radioiodination, with iodine-125, of a variety of carboxylic acids (24 examples). Such reactions were performed in different conditions and without the need of purifying the gold organometallic adduct (Figure 9b). In addition, to demonstrate the potential of our methodology, we will also present the radio-iodination of known radiotracers or iodinated drugs using the carboxylic acid function as a precursor. 

### 3.7. Mutational Analysis of the Surface Displayed HCN4 C-Linker-CNBD: A Flow Cytometry Based Ligand Binding Approach

WojciechowskiMagdalenaMaskriSarahKochOliverJoseJoachimInstitute of Pharmaceutical and Medicinal Chemistry, University of Münster, PharmaCampus, 48149 Münster, Germany; magdalena.wojciechowski@uni-muenster.de

**Figure 10:**
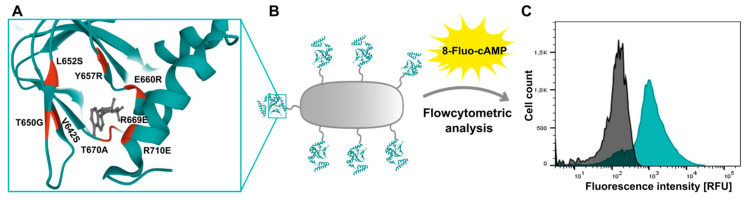
Schematic workflow representation. (**A**) Illustration of amino acids (red) mutated within the HCN4-CNBD with bound cAMP (PDB:3OTF). (**B**) *E. coli* cells presenting the HCN4-C-Linker-CNBD fusion protein on their surface were treated with 8-Fluo-cAMP followed by flow cytometric analysis. (**C**) Example histogram showing the fluorescence intensity of cells with bound (green) and unbound (black) ligand.

The hyperpolarization and cyclic nucleotide activated ion (HCN) channels are encoded by four genes. The channels gain increasing interest as they are associated with the development of various diseases e.g., epilepsy, bradycardia or neuropathic pain disorder. Due to their subtype dependent expression patterns, their function is not yet fully understood and requires further investigation. The ion channel properties such as voltage dependence, activation and closure kinetics are altered by the binding of endogenous cyclic nucleotides cAMP or cGMP to the cyclic nucleotide binding domain (CNBD) [53]. Here we analyse the impact of eight different amino acids within the CNBD on ligand binding using the method of Autodisplay [54] and flow cytometry [55]. The native HCN4 C-Linker-CNBD and the mutants were separately displayed as fusion proteins on the surface of *E. coli* cells. After incubation with 8-Fluo-cAMP, whole cell fluorescence was determined by flow cytometric analysis. Disturbed ligand binding caused by a mutation, resulted in decreased fluorescence intensity compared to the non-mutated C-Linker-CNBD, (Figure 10). 

It could be shown that mutations G660E, R669E and R710E led to an almost complete loss of whole cell fluorescence. This confirmed that these amino acids, supposed to directly interact with the ligand, are essential for binding [56]. Additionally, three amino acids not supposed to directly interact with the ligand were mutated. Nevertheless, the mutations V642S, L652S and Y657R led to a strong decrease of the fluorescence intensity. We showed here for the first time the impact of these residues on binding. Only moderate effects on ligand binding were observed for the mutant T670A and T650G. This ligand binding approach enables rapid investigation of residues essential for ligand binding without purification steps. 

### 3.8. Hit-to-Lead Optimization of a Novel RSV Small Molecule Entry Inhibitor

KoschChristina^1^GuneschAntonia P.^2^HaidSibylle^2^SakeSvenja^2^WagnerKonrad^1^SchererHugo^1^KanyAndreas M.^1^RoxKatharina^3^KieferAlexander F.^1^EmptingMartin^4^PietschmannThomas^2^HirschAnna K. H.^1^1Drug Design and Optimization (DDOP), Helmholtz Institute of Pharmaceutical Research Saarland (HIPS)–Helmholtz Centre for Infection Research, 66123 Saarbrücken, Germany; christina.kosch@helmholtz-hips.de2Institute for Experimental Virology, Twincore–Centre for Experimental and Clinical Infection Research, 30625 Hannover, Germany3PK/PD Unit, Helmholtz Centre for Infection Research (HZI), 38124 Braunschweig, Germany4Antiviral and Antivirulence Drugs (AVID), Helmholtz Institute of Pharmaceutical Research Saarland (HIPS)–Helmholtz Centre for Infection Research, 66123 Saarbrücken, Germany

Respiratory syncytial virus (RSV) is one of the main causes for acute lower respiratory tract infections (ALRI), threatening especially infants, immunosuppressed people, and the elderly [57,58,59]. Many entry inhibitors targeting the F protein have been discovered and optimized, but, by now, none of them has passed all clinical trials [60]. The only two FDA-approved drugs are Ribavirin and Palivizumab (Synagis). But, due to unfavorable side effects and no clear evidence of efficacy, the application of Ribavirin is not recommended. Therefore, treatment options are limited to Palivizumab, whose application has also some downsides: it is expensive and needs to be injected monthly [61,62]. 

This unmet medical need of alternatives encouraged us to initiate a multiparametric optimization campaign focusing on our highly active, non-cytotoxic RSV hit (IC50 45 ± 15 nm and CC50 > 100 µm), which we identified in a phenotypic screening of 42,000 substances. After hit confirmation by purity and chemical identity analyses as well as re-synthesis, we also analyzed its mode of action classifying the hit as an RSV entry inhibitor. To explore the compound’s structure–activity relationships (SARs) and optimize its physicochemical properties, we designed and synthesized around 150 derivatives with the support of a contract research organization (CRO). Their biological evaluation revealed–beside some interesting SAR insights–frontrunners of improved, single-digit nanomolar activity and no cytotoxicity up to 100 µm. Aiming at an inhalative application route, their profiling in terms of solubility, LogD7.4, permeability, and in vivo efficacy is currently ongoing. For further lead optimizations and to complete our SAR studies, we plan another synthetic round, co-crystallization experiments, and to perform a flexible alignment using Molecular Operating Environment (MOE). 

### 3.9. Terpenoid Hydrazones as Biomembrane Penetration Enhancers with Multi-Target Activity

NesterkinaMariia^1^KravchenkoIryna^2^HirschAnna K. H.^1^LehrClaus-Michael^2^1Department of Drug Design and Optimisation, Helmholtz Institute for Pharmaceutical Research Saarland–Helmholtz Centre for Infection Research (HZI), 66123 Saarbrücken, Germany; mariia.nesterkina@helmholtz-hips.de2Department of Drug Delivery across Biological Barriers, Helmholtz Institute for Pharmaceutical Research Saarland, 66123 Saarbrücken, Germany

Terpenoids are unique scaffolds that might be used for further chemical modification aimed at synthesizing compounds simultaneously affecting the central and peripheral nervous systems. Bearing this idea in mind, we designed and synthesized a series of terpenoid derivatives as potential analgesic and anticonvulsant agents that may additionally enhance the skin permeability [63]. The structure of terpenoid derivatives was characterized by ^13^C-NMR, ^1^H-NMR, FTIR-ATR, Raman-spectroscopy and ESI-mass spectrometry. All compounds were synthesized and purified up to 99% purity confirmed by high-performance liquid chromatography (HPLC); thermal behavior of terpenoid derivatives was performed by differential scanning calorimetry (DSC). 

**Figure 11:**
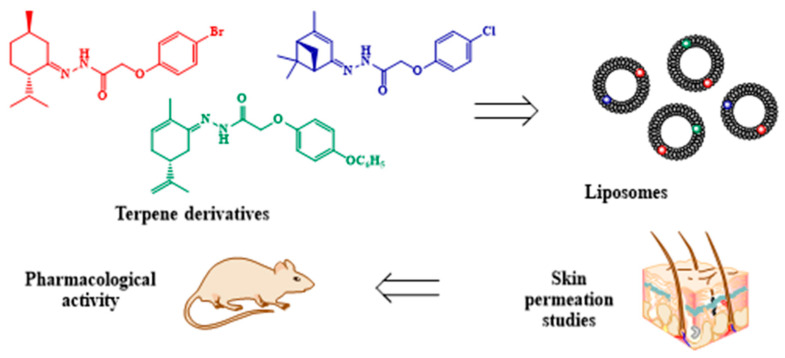
Terpenoid Hydrazones as Biomembrane Penetration Enhancers with Multi-Target.

The influence of terpenoid derivatives on the central and peripheral nervous system was reliably confirmed by evaluating their anticonvulsant and analgesic activity. The present findings indicate that all above-mentioned compounds possess antiseizure action throughout 24 h after oral administration on pentylenetetrazole-induced and maximal electroshock seizure (MES) convulsion models. The analgesic effect of compounds was elucidated after transdermal delivery via chemical-induced pain models using capsaicin and allyl isothiocyanate subplantar injection [64]. All the tested compounds were found to suppress painful sensation produced by noxious stimuli, indicating TRP channels (specifically, TRPV1 and TRPA1) as molecular targets of terpenoid derivatives. The mechanism of action of our terpenoid derivatives on phospholipids of artificial membranes and lipids isolated from the rat stratum corneum was studied by fluorescence probe technique and FT-IR spectroscopy. Thus, the current study implemented the idea for targeted synthesis of low molecular weight terpenoid derivatives followed by the investigation of their influence on molecular organization of the lipid matrix and further pharmacological testing (Figure 11). 

### 3.10. Aminobenzofuran-Containing Analogues of Proximicins B and C Exhibit Higher Antiproliferative Aactivity against Glioblastoma Cells Compared to Temozolomide in In vitro Cellular Assays

MadiehNasrin Shokrzadeh^1^AlquraynNorah Ahmed^2^TannaSangeeta^1^VaideanuAlexandra^2^SchatzleinAndreas^2^SinghHarprit^3^BrucoliFederico^1^1Leicester School of Pharmacy, De Montfort University, Leicester LE1 9BH, UK; p2507653@my365.dmu.ac.uk2UCL School of Pharmacy, University College London, 29/39 Brunswick Square, London WC1N 1AX, UK3Leicester School of Allied Health Sciences, De Montfort University, Leicester LE1 9BH, UK

The molecular framework of bacterial secondary metabolites has long been utilised for the development of antitumour drugs [65]. The marine environment has proven to be an excellent source of natural products and marine actinomycetes produce diverse types of secondary metabolites that have the potential to be progressed into therapeutic agents. Verrucosispora Fiedleri MG-37 [66] is a Gram-positive actinomycete isolated from deep-sea sediments that produce proximicin A, B and C. The distinctive structural element of proximicins is the 4-aminofuran-2-carboxylate unit forming a dipeptide core. Proximicins exhibit growth inhibitory activities against gastric adenocarcinoma (AGS) and hepatocellular carcinoma (Hep G2) [67]. Proximicin B induces apoptosis in both Hodgkin’s lymphoma (L1236) and T-cell leukaemia (Jurkat 16) cell lines, and proximicin C shows anti-proliferative activity against glioblastoma (U87 MG) and breast carcinoma (MDA-MD-231) [68].

In this project, we have synthesised and evaluated the biological activity of proximicin analogues containing a benzofuran moiety as the replacement of the di-furan scaffold of the parent compounds. Substitutions were introduced at both the N-terminus (NH_2_-terminus) and at the C-terminus (carboxyl-terminus) of the ethyl 5-amino benzofuran-2-carboxylate scaffold, which was synthesised from 5-nitrosalicylaldehyde and ethyl bromoacetate in high yield. We have prepared three libraries, in which the N-terminus was coupled with either heterocyclic carboxylic acids or methyl chloroformate, and the C-terminus was conjugated with a selection of aromatic amines including tyramine and tryptamine. The 35 novel compounds were purified and characterised by LC-MS, NMR spectroscopy and UPLC-HRMS. 

An MTT-based cell viability assay was conducted to evaluate the cytotoxic effects of these synthesised analogues against one human cancerous cell line (U-87 MG) and one human healthy cell line (WI-38). The compounds’ biological activities were compared to those of temozolomide, the chemotherapeutic agent of choice for the treatment of glioblastoma, was used as a reference control. Analysis of growth inhibitory concentrations values revealed that a number of benzofuran-containing proximicin analogues displayed higher antiproliferative activity against glioblastoma cells compared to temozolomide in U-87 MG, although exhibiting moderate levels of toxicity in WI-38 cells. 

## 4. Poster Presentations

### 4.1. Self-Assemblies of Azacitidine Prodrugs: A Promising Strategy of Treatment for Myelodysplastic Syndromes and Acute Myeloid Leukemia

BaroudMilad^1^LepeltierElise^1^El-MakhourYolla^2^ThepotSylvain^3^,^4^,^5^DuvalOlivier^1^,^3^1Micro & Nanomedecines Translationnelles (MINT), University of Angers, 49933 Angers, France; milad.baroud@univ-angers.fr2Environmental Health Research Lab, Faculty of Science, Lebanese University, 1700 Nabatieh, Lebanon3Department of Hematology, University Hospital of Angers, 49933 Angers, France4Federation Hospital of Universitaire Grand Ouest Acute Leukemia (FHU GOAL), 49933 Angers, France5Centre de Recherche en Cancérologie et Immunologie Nantes Angers (CRCINA), University of Angers, 49933 Angers, France

5-Azacitidine, a cytidine analogue used as a hypomethylating agent, is one of the main drugs for the treatment of myelodysplastic syndromes and acute myeloid leukemia in the elderly [69]. However, after administration, it exhibits several limitations, including restricted diffusion and cellular internalization due to its hydrophilicity, and a rapid enzymatic degradation by adenosine deaminase [70]. The aim of this study was to improve the drug cell diffusion and protect it from metabolic degradation via the synthesis of amphiphilic prodrugs and their potential self-assembly [71]. Azacitidine was conjugated to two different omega-3 fatty acids, eicosapentaenoic acid (EPA) and docosahexaenoic acid (DHA). The carboxylic acid group of the omega-3 fatty acids was effectively conjugated to the amine group of the azacitidine base, yielding two amphiphilic prodrugs that are uniquely cathepsin-B cleavable [72]. Nanoprecipitation of the obtained prodrugs was performed and self-assemblies were successfully obtained for both prodrugs, with a mean diameter of 190 nm, a polydispersity index below 0.2 and a positive zeta potential. The formation of self-assemblies was confirmed using pyrene as a fluorescent dye, and the critical aggregation concentrations were determined: 400 µM for AzaEPA and 688 µM for AzaDHA. Additionally, the stability of the obtained self-assemblies was studied and after 5 days their final stable arrangement was reached. Additionally, cryo-TEM revealed that the self-assemblies attain a multilamellar vesicle supramolecular structure. Moreover, the obtained self-assemblies were tested in vitro on HL-60 cell line (acute myeloid leukemia) presenting a promising cytotoxicity with a low IC_50_ value, comparable to that of free azacitidine.

### 4.2. Development of Novel Therapeutic Agents to Enhance Insulin Secretion

LenhamRhianna^1^MistryShailesh^2^LaughtonCharles^2^TurnerMark^1^1School of Science and Technology, Nottingham Trent University, Nottingham NG11 8NS, UK2School of Pharmacy, University of Nottingham, Nottingham NG7 2RD, UK; rhianna.lenham2020@my.ntu.ac.uk

**Figure 12:**
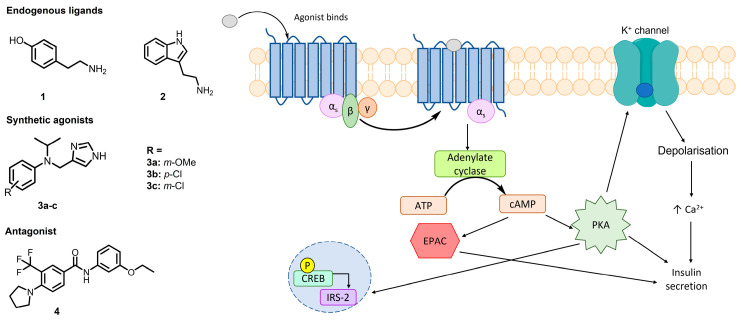
Structures of TAAR1 ligands (1–4) and the proposed therapeutic effects of activated pancreatic TAAR1. ATP; adenosine triphosphate, cAMP; cyclic adenosine monophosphate, EPAC; exchange proteins activated by cAMP, PKA; protein kinase A, CREB; cAMP response element-binding protein, IRS-2; insulin receptor substrate-2.

Defects in insulin action, secretion or both cause diabetes mellitus, a metabolic disorder affecting over 400 million people worldwide [73,74]. Several pharmacological agents have been developed to manage type 2 diabetes mellitus, however their effectiveness often declines over time. Trace amine-associated receptor 1 (TAAR1) is a G protein-coupled receptor expressed in several organs and cells including pancreatic β-cells [74]. Figure 12 shows pancreatic TAAR1 can amplify insulin secretion, thus it is recognised as a potential therapeutic target for novel oral hyperglycaemic drugs [75]. Research into developing TAAR1 agonists has revealed species-specific protein structure to be the main barrier for rational drug design. 

Currently, no structures of TAAR1 have been determined, therefore machine learning techniques were implemented for structure determination, with over 1000 potential models generated and ranked based on their ability to dock known TAAR1 ligands (Figure 12, 1–4) [76,77,78]. In parallel, three known human TAAR1 agonists (Figure 12, 3a–c, K_i_ = 4–6 nM) were resynthesized to validate the proposed pharmacology assays and to determine their insulin secretion ability [76]. Closely related novel compounds were designed and their pharmacological properties evaluated. The results obtained will support homology model development and future structure-activity relationship studies. As part of this communication, we will describe for the first time the synthetic routes along with the pharmacological response for these new molecules and the molecular docking studies to generate a human TAAR1 homology model. 

### 4.3. Design and Synthesis of Novel Fluorescent Allosteric Modulators for the Prostaglandin EP2 Receptor 

DaltonConstance^1^LaughtonCharles^1^HollidayNicholas^2^MistryShailesh N.^1^1Division of Biomolecular Science and Medicinal Chemistry, Division of Biomolecular Science and Medicinal Chemistry, School of Pharmacy, The University of Nottingham Biodiscovery Institute, University Park, Nottingham NG7 2RD, UK2School of Life Sciences, Queen’s Medical Centre, University of Nottingham, Nottingham NG7 2UH, UK; pcycd4@nottingham.ac.uk

**Figure 13:**
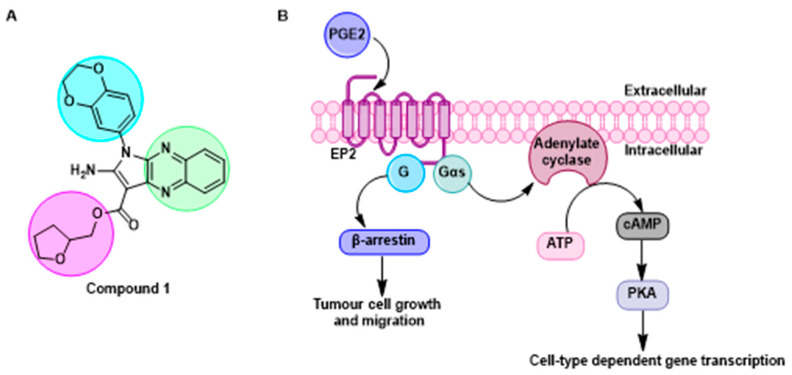
(**A**) Structure of Compound 1 highlighting three regions of interest for structural modification in blue, pink and green. (**B**) PGE2 binds and activates EPS, G_α__s_-mediated induction of adenylate cyclase to increase cytoplasmic cAMP levels. Downstream events are then mediated through protein kinase A. EP2 activation also induces β-arrestin which is known to promote tumor cell growth and migration.

The prostaglandin EP2 receptor (EP2) is a widely expressed G protein-coupled receptor activated endogenously by prostaglandin E2 (PGE2), which contributes to the development of chronic inflammation in cancer and has roles in diseases such as Parkinson’s, endometriosis, arthritis, intercranial aneurysms, glioblastoma and epilepticus (Figure 13B) [79,80]. EP2 antagonism is therefore considered a possible therapeutic approach to treat these diseases. Previously, numerous orthosteric antagonists (i.e., those that bind to the PGE2 binding site) have been synthesized [79,81]. In 2020, “Compound 1” (Figure 13A) was reported as the first allosteric EP2 antagonist that demonstrates a reversible, agonist dependent mode of action. 

As part of this communication, we will report our exploration of an expanded structure-activity-relationship dataset focusing on modifications at the tetrahydrofuran (pink), quinoxaline (green) and dioxane (blue—where most structural changes occurred in the literature) moieties. Initial work has identified a key intermediate in the synthesis of Compound 1, having a similar antagonist response when being pharmacologically characterised using a NanoBiT complementation assay against a known orthosteric antagonist [81].

### 4.4. Removing Cancer’s Immortality: The Design and Synthesis of Linear and Stapled Peptides Targeting the Dyskerin-Dyskerin PPI in Telomerase

WierSuzanne vanSearceyMarkBeekmanAndrewSchool of Pharmacy, University of East Anglia, Norwich NR4 7TJ, UK; s.van-wier@uea.ac.uk

One of the hallmarks of cancer is their ability to replicate limitlessly, making them immortal. In 80–90% of cancer cells this is due to the reactivation of telomerase, a protein complex which elongates telomeres at the end of chromosomes, protecting the chromosomes from degradation and preventing cell senescence. 

**Figure 14:**
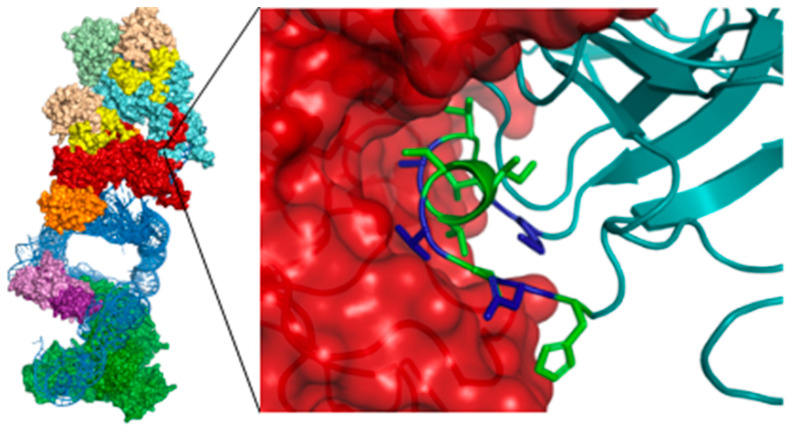
Left: Full telomerase structure, right: dyskerin-dyskerin PPI with derived peptide highlighted in green and the mutated residues commonly found in Dyskeratosis Congenita in this section highlighted in dark blue.

The Cryo-EM structure of telomerase published in 2021 provided an opportunity to identify new ways to target telomerase [82]. In patients with Dyskeratosis Congenita, a disease characterised by shortened telomeres, the structure showed that genetic mutations are transcribed to the dyskerin-dyskerin protein-protein interaction (PPI) in telomerase (Figure 14). In this project we aim to target this PPI, inhibiting the telomerase activity of cancer cells and thus removing cancer’s immortality. 

We will describe the design and synthesis of a peptide derived from the dyskerin sequence at this PPI and an alanine scan of this peptide to identify the amino acids most important for binding. These peptides were assessed for their α-helicity, showing low α-helicity, which was increased by 31-48% through hydrocarbon stapling. Current work is focused on the evaluation of the binding of these peptides to dyskerin and their effect on telomerase activity.

When compared to small molecule drugs, peptide therapeutics can have drawbacks such as their limited stability and cell permeability. Therefore, peptide-directed binding will be used to go from peptide to small molecule inhibitor by computationally identifying fragments able to replace parts of the peptide. This method has previously been applied successfully to quickly identify hit compounds for PPIs whilst minimising the organic synthesis and biological screening needed [17]. 

### 4.5. The Synthesis of 18β-Glycyrrhetinic Acid Derivatives which have Increased Antibacterial Activity against Staphylococcus Aureus

OdagiuMaria^1^RobertsAdam^2^WuPanpan^3^HongW. David^1^1Department of Chemistry, University of Liverpool, Liverpool, Merseyside L69 7ZD, UK2Centre for Drugs and Diagnostics, Department of Tropical Disease Biology, Liverpool School of Tropical Medicine, Liverpool, Merseyside L3 5QA, UK3School of Biotechnology and Health Sciences, Wuyi University, Jiangmen 529020, China; M.Odagiu@liverpool.ac.uk

**Figure 15:**
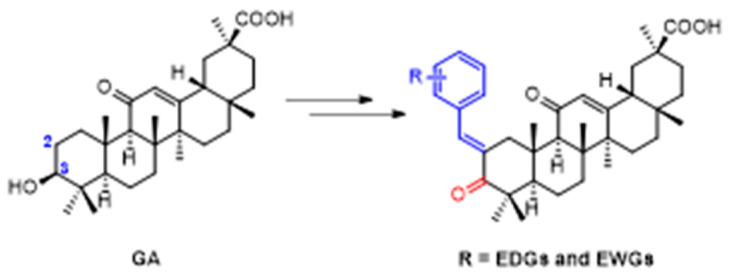
Structural variations of β-glycyrrhetinic acid (GA).

The natural pentacyclic triterpenoid 18 β-glycyrrhetinic acid (GA) is a minor component of liquorice root known to have a wide range of pharmacological effect. The antibacterial activity of this natural product is weak. However, studies have been shown that chemical modifications based on the GA scaffold could greatly improve its biological and physicochemical properties resulting in potent inhibition of pathogenic bacteria [83,84,85,86]. One of the most noticeable modifications is at the C-2 position with an addition of an aromatic side-chain that can considerably improve the antibacterial activity against *Staphylococcus aureus* spp. (Figure 15) [87]. 

To further explore the structure-activity relationship (SAR) of the side-chains at the C-2 position, a number of new analogues were designed and synthesised. The in vitro antibacterial activity of all the compounds was assessed against Gram-positive *S. aureus* and Gram-negative *E. coli*, using broth dilution, disc diffusion and bactericidal assays. The most potent compounds were found to have a MIC = 3.13 µM against *S. aureus* and MRSA, in comparison to an MIC of 200 µM for the starting material GA. These compounds were bactericidal with ~3–4 log reduction in bacterial numbers after 18 h incubation. The drug metabolism and pharmacokinetic (DMPK) properties of lead compounds were assessed in vitro to explore the druggability of this compound series. Further studies are being carried out to investigate the potential modes of action and resistance evolution using genomics and proteomics approaches. 

Antimicrobial resistance represents one of the most complex global challenges in this century and there is no doubt that the development of new antimicrobial therapeutics is urgently required. Therefore, the present findings on the modification of natural pentacyclic triterpene GA clearly shows its strong potential as a promising series of antibacterial agents in the fight against resistant pathogenic bacteria. 

### 4.6. Exploration of Intracellular Binding Pockets of CXC Chemokine Receptors for Allosteric Modulation

FrancisRhys^1^MistryShailesh^1^HollidayNicholas^2^LaughtonCharles^1^1School of Pharmacy, University of Nottingham, Nottingham NG7 2RD, UK; rhys.francis@nottingham.ac.uk2School of Life Sciences, University of Nottingham, Nottingham NG7 2TQ, UK

**Figure 16:**
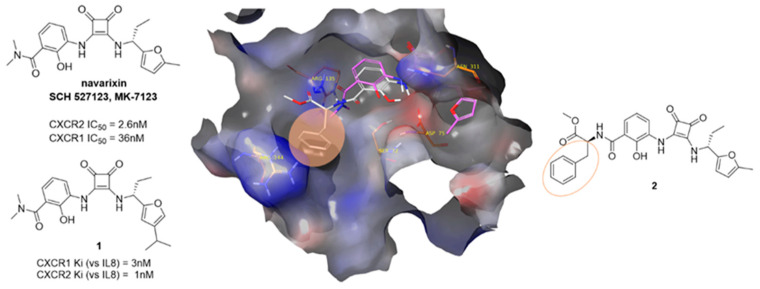
(left) Navarixin, currently the most advanced CXCR2 intracellular allosteric antagonist and (**1**) the most potent dual CXCR1/CXCR2 intracellular allosteric antagonist. (right) Proposed novel binding site extension between TM3 and TM5 in CXCR1. Overlayed docking of navarixin and proposed novel analogue (**2**) shows similar docking pose with the phenyl ring of the analogue buried in the receptor (highlighted orange).

Chronic inflammation is a major contributor to tumour growth. The CXC chemokine receptor (CXCR)/CXC ligand (CXCL) axis plays a vital role in the cancer microenvironment and is attributed to proliferation, angiogenesis, invasion, and metastasis [88,89]. Drug discovery programmes have attempted to target CXCR1/2 using small molecule therapeutic agents (navarixin and danirixin), however toxic side effects resulted in phase II clinical trials being terminated [90,91]. Mutation studies of CXCR2 have shown that the downstream signalling can be influenced by allosteric modulators binding to an intracellular site close to the G-protein binding region present in both CXCR1 and CXCR2 [92]. The most advanced class of compounds acting at this binding site are 3,4-diaminocyclo-3-ene-1,2-dione based antagonists (navarixin and 1, Figure 16) [93]. Development of novel antagonists can be aided by *in-silico* techniques however one of the barriers to rational drug design for CXCR1 antagonists is the limited structural data available (Figure 16). 

The aim of this work is to develop novel CXCR1 antagonists using a combination of in-silico and in-vitro methods. Molecular dynamic techniques and docking studies are being explored to construct and validate a homology model structure for the inactive, intracellular allosteric ligand bound state of CXCR1. Docking of known ligands for CXCR1 correlate with literature, validating the design and set a basis for further CXCR1 inhibitor design. Pocket and docking analysis have revealed a potential extension to the binding region between TM3 and 5. Extension of the previously described druggable binding pocket may play a key role in the design of a new class of CXCR1/2 antagonists. Ten 3,4-diaminocyclo-3-ene-1,2-dione based analogues were designed to probe this region and showed promising *in-silico* docking results. The series of analogues was subsequently synthesised and are under evaluation for activity at CXCR1. The *in-vitro* results obtained support the continued development of an accurate CXCR1 homology model along with structure-activity relationship studies for future drug design.

### 4.7. Base Mediated Radio-Iodination of Arenes from Organo-Silanes and-Germanes as Radiolabelling Precursors

BlouxHugo^1^HébertAlexandra^1^FabisFrédéric^1^CaillyThomas^1^,^2^,^3^,^4^1Normandie Université, UNICAEN, Centre d’Etudes et de Recherche sur le Médicament de Normandie (CERMN), 140000 Caen, France; hugo.bloux@unicaen.fr2IMOGERE, Normandie Université, UNICAEN 14000 Caen, France3Department of Nuclear Medicine, CHU Côte de Nacre, 14000 CAEN, France4Institut Blood and Brain @ Caen Normandie (BB@C), Boulevard Henri Becquerel, 14000 Caen, France

**Figure 17:**
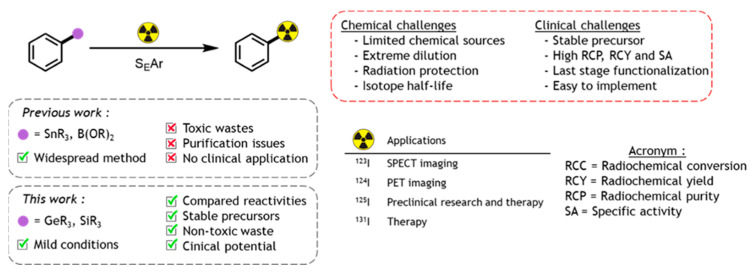
An overview of radio-iodination process.

The development of new radiolabelling strategies with iodine is crucial for multiple applications such as drug development, medical imaging or radiotherapy. Three different methods are already described for radio-iodination of arenes: SNAr, SEAr and the use of transition metal [51]. Widely described and widespread, ipso-radio-iododestannylation is very efficient but presents several chemical and toxicity drawbacks prohibiting clinical application. Silicon and germanium, belonging to the same group as tin, were also described in radio-iodination. Harsh acidic conditions are required when arylsilane precursors are used. Concerning organo-germane, only two publications were reported in the 1980’s. These functional groups, easily incorporated on the substrate, are more stable and less toxic than organo-tin. Determination of a new strategy for radio-iodination in mild conditions from organo-silanes or -germanes is a challenge will allow an alternative to the organotin precursor (Figure 17). Initially, 23 different (base) additives were evaluated. A radio-iodination set-up was assessed and the best conditions obtained were applied on more than 20 substrates. Finally, two molecules with therapeutic and imaging interest were formed using an organo-silane or -germane. 

### 4.8. A Marine Fungal Vanadium Haloperoxidase as an Alternative for Bromination of Electron-Rich Substrates

CochereauBastien^1^,^2^LokuhitigeSandamali^1^ChevéEmma^1^,^3^HollmannFrank^3^PouchusYves-François^1^Meslet-CladièreLaurence^2^RoullierCatherine^1^1Institut des Substances et Organismes de la Mer, Nantes Université, ISOMer, UR 2160, F-44000 Nantes, France; bastien.cochereau@etu.univ-nantes.fr2Université de Brest, INRAE, Laboratoire Universitaire de Biodiversité et Écologie Microbienne, F-29280 Plouzané, France3Department of Biotechnology, Delft University of Technology,2629HZ Delft, The Netherlands

Vanadium-haloperoxidases (vHPOs) are metalloenzymes which catalyze the synthesis of halogenated metabolites in vivo [94]. They usually activate C-H bonds by adding a chlorine, bromine or iodine atom to an electron rich substrate. First described in algae, they have rarely been observed in fungi [95,96]. Using genome mining approaches on different marine fungal strains, one gene of vHPO was detected in the filamentous black yeast *Hortaea werneckii* (UBOCC-A-208029). This enzyme, which is the second to be described in a marine fungal derived strain, was heterologously expressed in *Escherichia coli* BL21 (DE3). The recombinant, purified protein was biochemically characterized and found to possess higher affinity for bromine (Km = 26 µM) than for chlorine (Km = 237 mM). 

In the field of medicinal chemistry, halogen atoms can increase drug-target affinity, optimize pharmacokinetic parameters or increase stability of the biomolecular conformation by creating halogen-water-hydrogen bridges [97,98]. Therefore, the potential of HwvCPO for biocatalysis was evaluated. Organic solvent compatibility (such as EtOH, DMSO, DMF, ACN and MeOH) and comparison of kinetics and enzymatic reactivity with an already described enzyme, the vCPO from *Curvularia Inaequalis*, was performed. Bromination of small compounds like phloroglucinol, phenol and styrene were first investigated. Then, we moved on to more complex biological matrices such as fungal specialized metabolites and complex fungal extracts to increase their chemodiversity and produce new compounds with potentially new activities. Our first results using HwvCPO, allow us to propose this new enzyme as a new alternative eco-friendly tool to unspecifically halogenate molecules in vitro.

### 4.9. Emerging Role of Phospholipids and Lysophospholipids for Improving Brain Docosahexaenoic Acid as Potential Preventive and Therapeutic Strategies for Neurological Diseases

HachemMayssaNaciraHoudaDepartment of Chemistry, Khalifa University, Abu Dhabi 00971, United Arab Emirates; mayssa.hachem@ku.ac.ae

**Figure 18:**
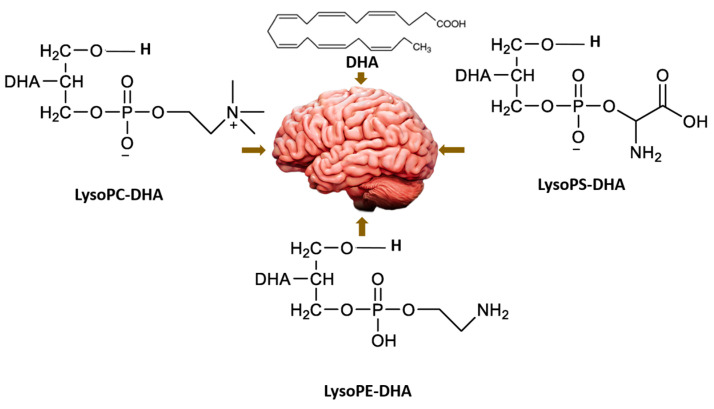
Targeting the brain with different DHA forms: free DHA, LysoPC-DHA, LysoPE-DHA and LysoPS-DHA.

Docosahexaenoic acid (DHA, 22:6n-3) is an omega-3 polyunsaturated fatty acid (PUFA) essential for neural development, learning, and vision. Although DHA can be provided to humans through nutrition and synthesized in vivo from its precursor alpha-linolenic acid (ALA, 18:3n-3), deficiencies in cerebral DHA level were associated with neurodegenerative diseases including Parkinson’s and Alzheimer’s diseases. The aim of this paper was to develop a complete understanding of previous and current approaches and suggest future approaches to target the brain with DHA in different lipid forms for potential prevention and treatment of neurodegenerative diseases [99]. We discussed the brain content and biological properties of phospholipids (PLs) and lysophospholipids (Lyso-PLs) with omega-3 PUFA focusing on DHA’s beneficial effects in healthy conditions and brain disorders. We emphasized the cerebral accretion of DHA when esterified at sn-2 position of PLs and Lyso-PLs, mainly lysophosphatidylcholine (LysoPC-DHA), lysophosphatidylethanolamine (LysoPE-DHA) and lysophosphatidylserine (LysoPS-DHA). Finally, we highlighted the importance of DHA-rich Lyso-PLs’ development for pharmaceutical applications since most commercially available DHA formulations are in the form of PLs or triglycerides, which are not the preferred transporter of DHA to the brain (Figure 18). 

In the context of prospective prevention and treatment of neurodegenerative syndromes, research on DHA benefits, as well as transport to the brain, has grown significantly in the last years. However, mechanisms on the bioavailability and DHA incorporation into the brain should be further explored. Although many studies have suggested that DHA is transported to brain by DHA transporters more specifically than others, future studies are required to explore a precise carrier molecule for improving brain DHA and this specific carrier might be used as a substitute in the prevention and treatment of neurodegenerative disorders. 

### 4.10. Playing the Serotoninergic Piano against Alzheimer’s Disease

GuiselinThomasLecouteyCédricRochaisChristopheDallemagnePatrickCentre d’Etudes et de Recherche sur le Médicament de Normandie, University of Caen Normandy, 14032 Caen, France; thomas.guiselin@unicaen.fr

Alzheimer’s disease (AD) has been described for the first time more than a century ago and its molecular causes are now the subject of a relative consensus around the hyperphosphorylation of the TAU protein and the aggregation of the ß-amyloid (Aß) peptide into neurotoxic oligomers. 

**Figure 19:**
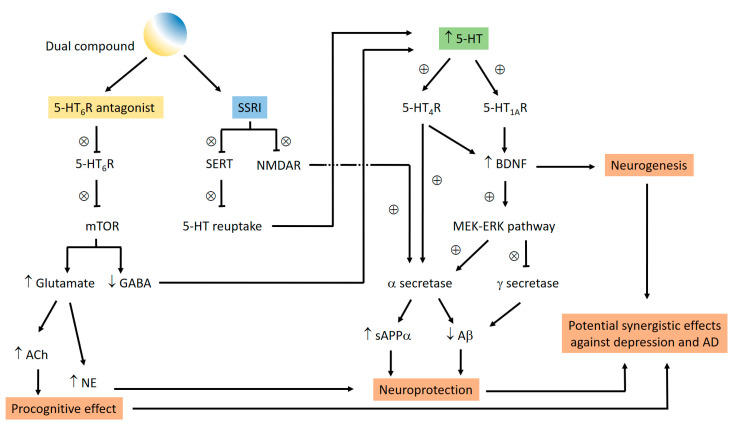
Hypothetical mechanisms of a dual SSRI/5-HT_6_R antagonist.

5-HT_6_R is a valuable target of therapeutic interest in AD. The inactivation leads, in particular, by inhibition of the mTOR pathway, to procognitive effects observed in rodents (Figure 19). 5-HT_6_R antagonists are also able to promote, via a decrease of GABA levels, the 5-HT concentration in the brain. As a result, 5-HT_6_R antagonists have shown positive effects against memory disorders affecting these animals. The serotonin transporter (5-HTT), on the other hand, has also demonstrated its interest as a target in the treatment of AD. Indeed, its antagonists, which selectively inhibit serotonin reuptake (SSRI) at the pre-synaptic level have also demonstrated their ability to reduce, after chronic administration, amyloid deposition and senile plaques in transgenic AD mice and also in humans. The increase in serotonin levels, that they otherwise induce in animal models of AD, allows them to exert a positive effect on the memory disorders these animals suffer. 

The objective of this project is to design a pleiotropic drug candidate both able to inhibit 5-HT reuptake and selectively to antagonize 5-HT_6_R (Figure 19). To our knowledge, such a compound has never been described before. 

Starting from a hit, selected from CERMN’s chemical library, a first set of derivatives has been synthesized in order to establish the structure-activity relationships and improve the expected activities. 

### 4.11. Innovative Pleiotropic Prodrugs with Potential Therapeutic Interest in Down Syndrome and Alzheimer’s Disease

WangAliceSinceMarcDavisAudreyDallemagnePatrickRochaisChristopheCentre d’Etudes et de Recherche sur le Médicament de Normandie (CERMN), UNICAEN, 14000 Caen, France; alice.wang@unicaen.fr

**Figure 20:**
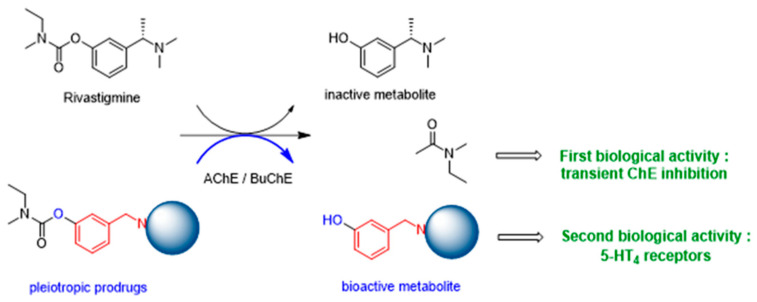
Mechanism of Action of rivastigmine and pleiotropic prodrugs.

People with Down syndrome (DS) have a higher risk of developing dementia such as Alzheimer’s disease (AD) from the age of 40. These diseases are multifactorial and studies have shown a link between AD and DS [100]. However, the association of several drugs can cause additional side effects, pharmacokinetic and drug-drug interaction problems. Thus, to avoid these occurrences, Multi-Target Directed Ligands (MTDLs) approach seems promising for the development of new drugs against these diseases [101]. In this context, the design of novels MTDLs, carbamoyl prodrugs capable of inhibiting cholinesterases (AChE or BuChE) [102,103] according to the same mechanism as rivastigmine [104] and then releasing an active metabolite to reach 5-HT_4_ receptors [105] is a promising way to treat AD (Figure 20). Four novel compounds were synthesized, tested and have shown interesting activity, in the nM order, on AChE, BuChE and 5-HT_4_ receptors. These promising results allow us to design other pleiotropic prodrugs to reach other targets involved in DS and AD. 

### 4.12. Drug Design and Biological Evaluation of MT5-MMP (Membrane-Type 5 Matrix MetalloProteinase) Inhibitors for a New Potential Treatment of Alzheimer’s Disease

RémondinChloéDavisAudreyBureauRonanRochaisChristopheDalPatrickCentre d’Etudes et de Recherche sur le Médicament de Normandie, Normandie University, Université de Caen Normandie,14000 Caen, France; chloe.remondin@unicaen.fr

**Figure 21:**
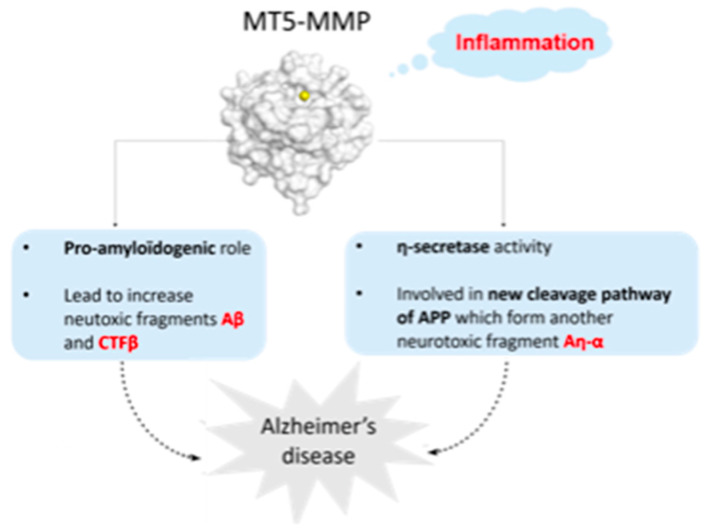
Presentation of MT5-MMP’s activities and APP processing [107].

Alzheimer’s Disease (AD) is the most common form of senile dementia in the world and is a main socio-economic problem in health care. The appearance and progression of this neurodegenerative disease are associated with the aggregation of the β-amyloid peptide (Aβ). A therapeutic strategy against AD could consist in the development of molecules able to interfere with specific steps of Aβ aggregation. However, AD is a multifactorial disease and several other targets are implied in its pathogenesis. One of these targets, recently discovered, is MT5-MMP [106], a metallo-enzyme which has two main deleterious activities in brain (Figure 21). MT5-MMP plays a proamyloidogenic role and promotes the formation of neurotoxic peptides (Aβ, CTFβ). Further MT5-MMP exerts also a η-secretase activity and cleaves APP resulting in a newly discovered neurotoxic fragment named Aη-α. In consequence, the inhibition of MT5-MMP could be another therapeutic strategy against AD. With the aim to confirm this new therapeutical approach, this work is beginning to develop selective inhibitors with good activity. 

### 4.13. Controlling Coiled Coils: Harnessing Transcription Factors with Protein-Protein Interactions

HydeEllieHobsonEmilyGoddardZoëO’ConnellMariaSearceyMarkBeekmanAndrewSchool of Pharmacy, University of East Anglia, Norwich NR4 7TJ, UK; ellie.hyde@uea.ac.uk

The Nrf2-MafG protein-protein interaction (PPI) leads to the transcription of cytoprotective genes in the body [108]. However, cancer cells can hijack this pathway to create chemoresistance [109]. Designing new, more potent inhibitors of the Nrf2 pathway will provide an invaluable research tool with the potential for producing a new chemo-sensitizing agent for cancer treatment. 

**Figure 22:**
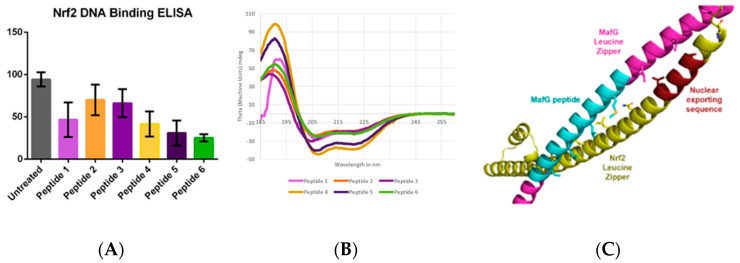
(**A**) Nrf2 DNA Binding ELISA presented as a percentage over vehicle control; (**B**) Circular Dichroism of Peptides 1-6 in 50:50 TFE: Potassium Phosphate Buffer; (**C**) AlphaFold model of the Nrf2-MafG PPI.

Nrf2 and MafG are the transcription factors that control the cytoprotective response. These proteins bind via alpha helical structures that dimerise through the formation of a coiled coil. Coiled coils make a fascinating type of PPI as they feature a repeated motif throughout the alpha helix, [110,111] allowing us to easily predict the positions of the amino acids essential for binding and those that can be altered to improve stability [112]. The protein-protein interactions between Nrf2 and MafG were predicted using AlphaFold [113]. This has allowed us to identify a peptide sequence mimicking MafG that could disrupt Nrf2-MafG binding, _76_KEELEKQKAELQQEVEKLASENASMKLE_104_ (Figure 22C).

This presentation will describe the design and synthesis of peptides which bind to Nrf2 and control the MafG interaction, including analysis using Nrf2 DNA binding ELISA (Figure 22A), downstream Nrf2 activity assays and circular dichroism (Figure 22B).

This work is funded by the UKRI BBSRC as a part of the Norwich Research Park Doctoral Training Partnership.

### 4.14. Influence of Aromaticity on the Covalent Inhibition of Nafamostat Derivatives as Potential SARS-CoV-2 Antiviral Agents

FarkasBarbaraMinneciMarcoRozasIsabelTrinity Biomedical Sciences Institute, Trinity College Dublin, D07 R590 Dublin, Ireland; FarkasB@tcd.ie

Outbreak of the Severe Acute Respiratory Syndrome Coronavirus 2 (SARS-CoV-2) in 2019 has emerged as a global health crisis. Despite the enormous efforts devoted to developing suitable treatments, to date there are only three clinically approved small drug for treating SARS-CoV-2 (remdesevir, molnupiravir and paxlovid), showing limited specific efficacy. Transmembrane protease serine 2 (TMPRSS2) was identified as a key host cell factor for viral entry and pathogenesis of SARS-CoV-2 [114] and it is an attractive small drug target since blocking the host cell receptors was found to have beneficial effects of diminished drug resistance compared to direct virus targeting. Additionally, repurposing of approved inhibitors for a specified target is a valuable time- and cost-effective design strategy when detecting antiviral candidates against new viruses, and serine protease inhibitors have long been of interest within research community. 

**Figure 23:**
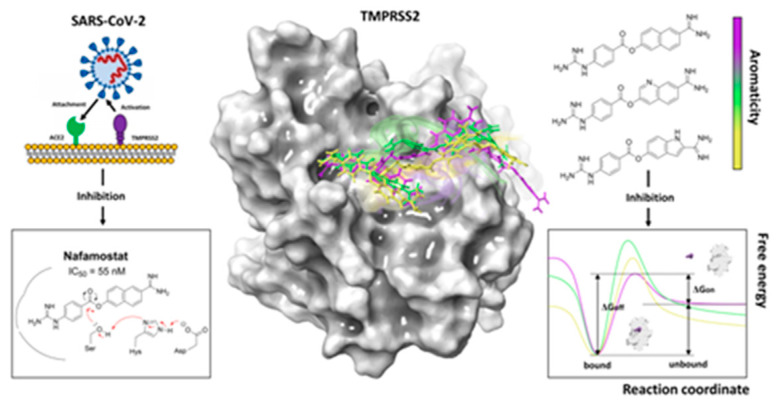
Scheme of TMPRSS2 targeting by nafamostat-based derivatives for potential SARS-CoV-2 inhibition.

However, known TMPRSS2 inhibitor nafamostat, whose covalently co-crystallised x-ray structure was recently published, was found to have a short life-time in the lung, which is a focal point of SARS-CoV-2 viral spread [115]. Re-design attempts for covalent inhibitors usually aim to both optimise the secondary non-covalent interactions between the ligand and the protein, as well as to fine tune the structure of the warhead to achieve optimal reactivity upon covalent binding. However, impact of the scaffolds not directly included in the covalent bond formation on the reaction barriers is often overlooked. This work investigates computationally the aromaticity effects of non-covalent scaffolds of nafamostat derivatives on binding affinities and covalent binding energy barriers within TMPRSS2 target in the quest of identifying new factors for developing potential SARS-CoV-2 antiviral agents (Figure 23). 

### 4.15. Identification of Heterocyclic Aminothiadiazoles as Analogues of Fast Acting 1,3,4-Oxadiazole Based Antimalarials

QieLi^1^HongW. David^1^LeungSuet C.^1^BrowneFrancesca E.^1^Lloyd-HughesRhoslyn^1^HowardOlivia^1^TaramelliDonatella^2^O’NeillPaul M.^1^1Department of Chemistry, University of Liverpool, Liverpool L69 7ZD, UK; Qie.Li@liverpool.ac.uk2Department of Pharmacological and Biomolecular Sciences, University of Milan, Via Pascal 36, 20133 Milan, Italy

Malaria is a mosquito-borne infectious disease that remains a global health problem. It was reported by the World Health Organization that 241 million cases resulted in 627,000 deaths in 2020 [116]. *Plasmodium falciparum* (*Pf*) and *Plasmodium vivax* are the two main kinds of malaria parasites that infect humans, with the former the most important variant in terms of mortality. The emergence of parasite drug resistance to artemisinin-based combination therapies, the current first-line treatment strategy, is retarding the progress of malaria control and elimination. Additional antimalarial medicines with novel mechanism of actions are a primary focus of drug discovery efforts. 

GSK developed 2-amino-1,3,4-oxadiazoles, a promising non-endoperoxide scaffold for novel antimalarials, from a screen hit of the Tres Cantos Antimalarial Set [117]. However, this kind of aminoxadiazole derivatives has the capacity to generate genotoxic metabolites such as hydrazine via Phase I metabolism in vitro and in vivo. We hypothesised that this genotoxicity alert could be mitigated by using appropriate bioisosteres to replace the problematic oxadiazole in the scaffold, whilst maintaining the antimalarial properties and further improving the physicochemical and DMPK properties. 

**Figure 24:**
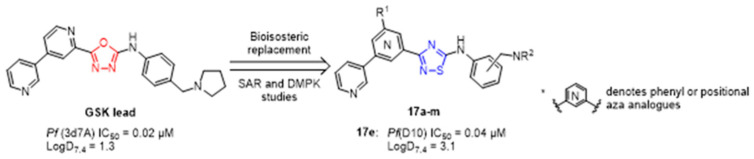
Synthesis of aminothiadiazole analogues **17a-m**.

A series of aminothiadiazole analogues were identified in which **17e** showed good antimalarial activity and excellent water solubility with promising DMPK profiles comparing to the original lead (Figure 24). It exhibited reasonable AUC and bioavailability in the further in vivo PK study and showed its developability as a potent fast acting antimalarial candidate with balanced drug-like properties. 

The authors thank AstraZeneca for DMPK predication and Measurement. 

### 4.16. Design and Synthesis of 12-Thiazole Abietanes as Selective Inhibitors of the Human Metabolic Serine Hydrolase hABHD16A

MoreiraVânia M.^1^,^6^,^7^AhonenTiina J.^1^NgChoa^2^SavinainenJuha R.^3^Yli-KauhaluomaJari^1^KalsoEija^4^,^5^LaitinenJarmo T.^3^GreavesJennifer^2^1Drug Research Program, Division of Pharmaceutical Chemistry and Technology, Faculty of Pharmacy, University of Helsinki, FI-00014 Helsinki, Finland; vmoreira@ff.uc.pt2Centre for Sport, Exercise and Life Sciences, Coventry University, Coventry, UK3School of Medicine, Institute of Biomedicine, University of Eastern Finland, FI-70211 Kuopio, Finland4Department of Pharmacology, Faculty of Medicine, University of Helsinki, FI-00014 Helsinki, Finland5Department of Anaesthesiology, Intensive Care and Pain Medicine, Helsinki University Hospital and University of Helsinki, FI-00014 Helsinki, Finland6Centre for Neuroscience and Cell Biology (CNC), University of Coimbra, Portugal and Centre for Innovative Biomedicine and Biotechnology (CIBB), University of Coimbra, Portugal7Laboratory of Pharmaceutical Chemistry, Faculty of Pharmacy, University of Coimbra, Portugal

Neuroinflammation, defined as inflammation of tissue within the peripheral and central nervous system, has been implicated in the development of chronic pain via sensitization of nociceptive neurons [118,119]. Therefore, identifying and targeting the processes and molecules involved in neuroinflammation is regarded as an effective strategy for innovative chronic pain treatments. In this regard, the metabolic serine hydrolase ABHD16A, belonging to the ABHD (α,β-hydrolase domain) enzyme family may potentially be a novel key target in inflammation-mediated pain [120,121]. Selective inhibitors of hABHD16A (human ABHD16A) have not yet been reported. 

In the screening of an in-house library of compounds, we have identified 12-thiazole abietanes as a new class of reversible inhibitors of the human metabolic serine hydrolase [122]. Upon the optimization of the first hit compound we discovered a 2-methylthiazole derivative with an IC_50_ value of 3.4 ± 0.2 µM and promising selectivity towards ABHD16A. Our current work focuses on screening a further series of 12-thiazole abietanes on ABHD16A. Our study suggests abietane-type diterpenoids present an attractive starting point for the design of selective ABHD16A inhibitors, contributing towards understanding the significance of hABHD16A inhibition in vivo. 

### 4.17. 8-Substituted 2,3-Diarylimidazo [1,2-a]Pyrazines as Casein Kinase 1 Inhibitors Endowed with Antileishmanial Activity

TisseurLhana^1^BazinMarc-Antoine^1^CojeanSandrine^2^PagniezFabrice^1^BernadatGuillaume^2^LogéCédric^1^CavéChristian^2^Ourliac-GarnierIsabelle^1^PicotCarine^1^MorgadoCathy^1^LeclercqOlivier^3^BaratteBlandine^4^RachidiNajma^3^BachStéphane^4^LoiseauPhilippe^2^PapePatrice Le^1^MarchandPascal^1^1Nantes Université, Cibles et médicaments des infections et de l’immunité, IICiMed, UR 1155, F-44000 Nantes, France; lhana.tisseur@etu.univ-nantes.fr2Université Paris-Saclay, Chimiothérapie Antiparasitaire, BIOmolécules: Conception, Isolement et Synthèse–BioCIS UMR 8076 CNRS, F-92296 Châtenay-Malabry, France3Sorbonne Universités, UPMC Paris 06, CNRS USR3151 “Protein Phosphorylation and Human Disease” group, Station Biologique, F-29680 Roscoff, France4Institut Pasteur and INSERM U1201, Unité de Parasitologie Moléculaire et Signalisation, F-75015 Paris, France

**Figure 25:**
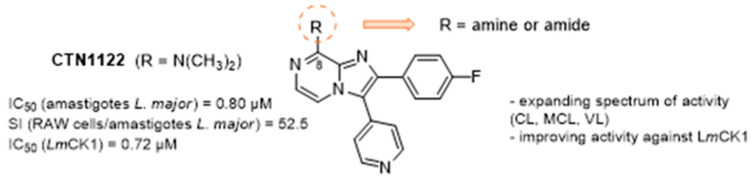
Modulation from the hit compound **CTN1122**.

Leishmaniasis–visceral leishmaniasis (VL), cutaneous leishmaniasis (CL) and mucocutaneous leishmaniasis (MCL)–are classified as one of the 20 so-called neglected tropical diseases of the WHO program [123]. These parasitic diseases are endemic in nearly 100 countries worldwide and constitute a serious public health problem with 12 million people infected, 350 million others at risk of infection and 40,000 deaths each year. In 2012, mainly due to global warming, visceral leishmaniasis (VL) was declared as a new emerging disease in Europe. Current treatments–pentavalent antimonials, amphotericin B, miltefosine, pentamidine, paromomycin and sitamaquine [124]–are toxic, costly and lead to the development of parasite resistance. Therefore, it’s important to rapidly develop new and more effective drugs with new mechanisms of action to resolve this emerging resistance. Previously, we reported the discovery of CTN1122 [125], an imidazo [1,2-*a*]pyrazine derivative with promising antileishmanial properties and targeting a protein of interest: *Leishmania* Casein Kinase 1 paralog 2 (*L*-CK1.2) [126]. In this context, we decided to synthesize CTN1122 analogues (Figure 25) in order to improve the pharmacological activity profile.

Eighteen new analogues resulting from the optimization of CTN1122, by the modification of the substituent in position 8 of the imidazo [1,2-*a*]pyrazine ring, were obtained. The study of these analogues will allow to discuss the structure-activity relationship regarding their antileishmanial properties, their *Lm*CK1 target protein inhibition capacities and taking into account their toxicity profile [127].

### 4.18. 2-Prenylated Benzopyrans with PPARα/γ Agonist Activity as Potential Drugs for Metabolic Syndrome

GarcíaAinhoa^1^Villarroel-VicenteCarlos^1^,^2^VilaLaura^1^,^2^BernabeuÁlvaro^1^HennuyerNathalie^3^StaelsBart^3^CabedoNuria^1^,^2^CortesDiego^1^1Department of Pharmacology, Faculty of Pharmacy, University of Valencia, 46100 Valencia, Spain; ainhoagarcia05@gmail.com2Institute of Health Research-INCLIVA, Hospital Clínico Universitario de Valencia, 46010 Valencia, Spain3Université de Lille, Inserm, CHU Lille, Institut Pasteur de Lille, U-1011-EGID, F-5900 Lille, France

**Figure 26:**
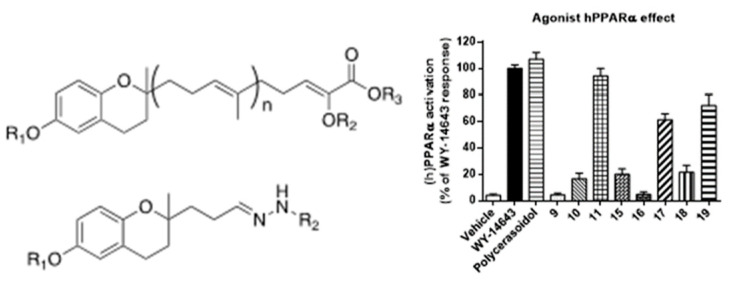
Left: General structure of prenylated benzopyrans. Right: PPARα activity.

Peroxisome proliferator activated receptor (PPARs) are nuclear receptors activated by ligands which are implicated in glucose, lipid methabolism and inflammation. Series of 2-prenylated benzopyrans have been synthesized as analogues of the natural polycerasoidol, a dual PPAR α/γ agonist with anti-inflamatory effects [128]. The prenylated side chain varies in the different series and consists of an a-alkoxy-a,b-unsaturated ester moiety (series 1 and 2) or a hidrazine substituent (series 3) (Figure 26). In series 1 and 2, prenylation was introduced by the Horner-Wads-worth-Emmos reactions [129] while in series 3 has undergone an amination reaction. Synthetic derivates showed high or moderate efficacy to activate both hPPARα and hPPARγ as a dual PPAR α/γ agonists. These prenylated benzopyrans emerge as lead compounds potentially useful for preventing metabolic syndrome. 

This work was funded by grants PFIS (FI19/00153) to C.V.V and Miguel Servet programme (CPII20/00010) to N.C. from the Carlos III Health Institute (ISCIII) co-funded by the European Social Fund (ESF) “Investing in your future”, and by grants PI18/01450, PI21/02045, AICO/2021/081 and APOTIP/2020/011 from the GVA, ISCIII and co-funded by European Regional Development Fund (ERDF).

### 4.19. A Novel Approach to Traceless Generation of Serine Protease Inhibitors under Bioassay-Compatible Conditions

VuLan PhuongZyulinaMariaHingstAlexandraGütschowMichaelPharmaceutical Institute, Pharmaceutical & Medicinal Chemistry, University of Bonn, 53121 Bonn, Germany; lanphuong.vu@uni-bonn.de

Dynamic combinatorial chemistry has emerged as a powerful strategy to identify hit compounds for biological targets and gained significant impact on drug discovery. The application of combinatorial chemistry science has also revolutionized high-throughput screening technologies, as well as chemical lead optimization [130].

**Figure 27:**
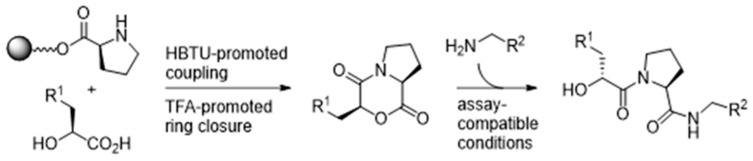
A Novel Approach to Traceless Generation of Serine Protease Inhibitors.

We applied a combinatorial method for the design and identification of inhibitors of therapeutically relevant serine proteases, also referred to as substrate-analogue inhibitors [131], such as thrombin and factor Xa. Imino acid derived diketomorpholines were conceptualized as generally applicable key intermediates prepared through solid-phase synthesis. Due to their susceptibility to ring cleavage upon treatment with primary amines [132], these diketomorpholines were appropriate educts for a traceless generation of bioactive compounds (Figure 27). This approach led to a compound library whose members were prepared under bioassay-compatible conditions and can directly subjected to the in situ evaluation, allowing a fast prediction of hit compounds. Highly active inhibitors for serine proteases of the coagulation cascade have been identified [133]. 

### 4.20. PROTAC Technology: A New Opportunity to Target Proteins Overexpressed in Chemoresistant Ovarian Cancer

CornuMarie^1^GuedeneyNicolas^1^PaysantHippolyte^2^KieffetCharline^1^PoulainLaurent^2^Voisin-ChiretAnne Sophie^1^1Centre d’Etudes et de Recherche sur le Médicament de Normandie (CERMN), Normandie Université (UNICAEN), 14000 Caen, France; email marie.cornu@unicaen.fr2Inserm U1086 ANTICIPE, Normandie Université (UNICAEN), 14000 Caen, France

**Figure 28:**
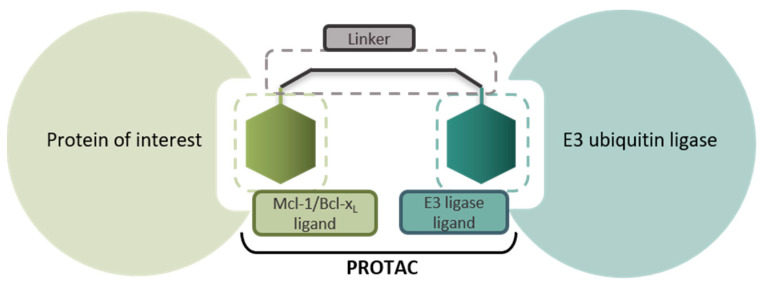
Structure of our PROTAC molecule.

Delayed diagnosis combined with the resistance of cancer cells to chemical treatments make ovarian cancer the deadliest gynecological cancer. Evading apoptosis of cancer cells is one of the causes of this drug resistance. It has been demonstrated that this escape is due to the overexpression of two proteins in particular: Mcl-1 and Bcl-xL [134]. However, the inhibition of these proteins leads to both a cardiac (for Mcl-1) and a platelet (for Bcl-xL) toxicity. In addition, inhibition of only one of the two proteins induces an overexpression of the other. The objective of this project is to synthesize molecules with a dual activity, that is to say having the ability to simultaneously degrade Mcl-1 and Bcl-xL these two proteins [135]. For this, compounds were synthesized according to the PROTAC (PROteolysis Targeting Chimeras) approach. Developed in 2001 by Prof. Crews [136] and his team, it creates heterobifunctional molecules recruiting target proteins on one hand and an E3 ligase on the other hand, linked together by a linker whose length and nature can vary. Once the ternary complex has been established, the proteins will be segmented into amino acids thanks to the ubiquitin-proteasome system engaged by the E3 ligase (Figure 28).

Concomitant degradation between Mcl-1 and Bcl-x_L_ could restore balance in cancer cells and then the programmed cell death, called apoptosis. In that way, a first PROTAC molecule was synthesized with, on the first side, an analog to pyridoclax [137] a known inhibitor of Mcl-1, as the target protein recruiting ligand, and on the other side, a ligand can recruit type CRBN ligases. In vitro evaluated on chemoresistant cancer cell lines by Dr. Poulain’s team, this PROTAC has shown encouraging results. The PROTAC strategy has opened new avenues in terms of drug design and chemical biology; although PROTAC technology has a promising future in drug development, it also has many challenges [138].

### 4.21. Novel Fluorescent Porphyrin Probes to Target G-Quadruplexes

StipaničevNikolinaRozasIsabelTrinity Biomedical Sciences Institute, School of Chemistry, Trinity College Dublin, The University of Dublin, 152-160 Pearse St., D02 R590 Dublin, Ireland; e-mail: rozasi@tcd.ie

Various molecules have been synthesized as probes to target G-Quadruplexes (G4s); however, the lack of selectivity for binding to G4s over double-stranded DNA (dsDNA) as well as binding to a particular G4 over others remains being a challenge [139,140]. Taking the topology of targeted G4s into account is essential for the design and fine-tuning of G4 probes [141]. In this project we aim to design, synthesize and biophysically study new conjugates of porphyrins and diaryl guanidine DNA binders as fluorescent probes that bind selectively to G4s. The computational studies carried out using different G4 3D structures from the Brookhaven Protein Databank as templates, have shown that the porphyrin moiety stacks on top of the upper guanine-tetrad whereas the diaryl guanidinium moiety is responsible for the interaction between the ligand and a side groove of the G4s [30]. Accordingly, a new family of flexible linked conjugates are being prepared and their binding to the G4s and dsDNA is being studied by biophysical methods. Thus, the results of UV-thermal melting and molecular docking studies of the new derivatives (flexible linker) and the previously prepared porphyrin conjugates with a semi-flexible linker are compared and analyzed. 

Acknowledgement: This work is supported by a postgraduate scholarship of the Irish Research Council (GOIPG/2018/2336). 

### 4.22. Synthesis and Physicochemical Properties of Amino Acid Prodrugs of the Radiosensitser Pyrazinib

QaisarAlina^1^O’SullivanJacintha^2^O’BoyleNiamh M.^1^1School of Pharmacy and Pharmaceutical Sciences, Trinity College Dublin, Dublin, Ireland. email: qaisara@tcd.ie2Department of Surgery, Trinity Translational Medicine Institute, St. James’s Hospital, Trinity College Dublin, Dublin, Ireland

**Figure 29:**
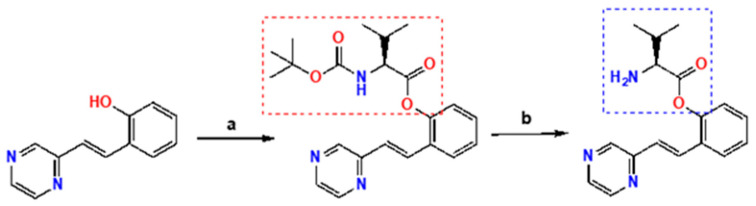
Reagents and conditions: (**a**) N-Boc-valine, HBTU, DIPEA, anhydrous acetonitrile, room temperature, 24 h (**b**) DCM, TFA, room temperature, 1 h.

Successful drugs must overcome many hurdles on their journey to clinical use, and it is essential to have a favorable solubility and stability profile. We aim to improve the solubility of a promising anti-cancer agent, pyrazinib, which is under development as an adjunct treatment for oesophageal adenocarcinoma (OAC). OAC is an aggressive disease with 5-year survival rates of <20%. Pyrazinib was identified as a radiosensitiser which showed anti-angiogenic and anti-metabolic activity in-vivo in zebrafish and in-vitro isogenic models of OAC radioresistance [142]. However, extremely poor water solubility of pyrazinib limits the options for in vivo testing and delivery to patients. This study aims to overcome solubility issues by synthesis of pyrazinib prodrugs. Prodrug formation is an excellent option for improving the solubility of drugs. We have synthesised a series of amino acid prodrugs of pyrazinib using HBTU-assisted coupling (example of valine showed in Figure 29). This has improved the water-solubility and will enable further progression of the compound along the drug delivery pipeline into clinical studies.

All synthesised compounds were purified by flash column chromatography and chemically characterised by techniques such as NMR (^1^H and ^13^C), FTIR spectroscopy, HRMS and melting point analysis. The radiosensitising and anti-metabolic activity of the series of pyrazinib amino acid prodrugs in in vitro isogenic models of OAC radioresistance (OE33P and OE33R cells) will be compared to pyrazinib. 

Acknowledgements: This work is supported by the Trinity College Dublin 1252 Postgraduate Scholarship and the Faculty of Health Sciences Dean’s Research Initiatives Fund.

### 4.23. The Synthesis of DSA Analogues for the Treatment of Cancer

CassidyLilyBeekmanAndrewSearceyMarkSchool of Pharmacy, University of East Anglia, Norwich Research Park, Norwich NR47 TJ, UK; Lily.Cassidy@uea.ac.uk

Cancer is the second leading cause of death in the world, with more than one in two people being diagnosed within their lifetime [143]. Cancer can be treated through different means including chemotherapy, the choice of drug depends on the type of cancer and the extent of its spread. 

The duocarmycins are natural compounds that are highly potent DNA alkylating agents. They bind to the minor groove in AT rich regions of DNA and are among the most potent cytotoxic compounds known, offering a great potential for cancer treatment. However, high toxicity and low selectivity mean there has been limited progress of duocarmycins as chemotherapeutic agents. One approach to achieve selective cytotoxicity is with antibody drug conjugates, or ADCs. Here the cytotoxic drug is bound by a linker to the antibody, in doing this the payload can be targeted directly to the tumours. A duocarmycin based ADC called SYD985 is in phase III clinical trials for the treatment of HER-2 Metastatic Breast Cancer [144]. This shows an exciting new field to be explored, this project will look at preparing a dimeric payload that could potentially be used as a warhead for an ADC. 

The duocarmycin family consists of different alkylating subunits, including the duocarmycin SA unit, DSA, one of the most potent subunits and of great interest in the design of analogues [145].

This project follows work previously done by Searcey where DSA was synthesised with an appropriate protection strategy. In doing this, analogues can be made using solid phase synthesis techniques already established within the research group. This SPS method allows a library of analogues to be made and studied, with focus on making dimers. Duocarmycin payloads in ADCs have shown promise as dimers with dimeric seco-CBI payloads and PBD dimers both in clinical trials. Using our approach we will be able to “tune” the physicochemical characteristics of the dimers by varying amino acid-based linker structures.

### 4.24. Synthesis of Prenylated Xanthones as Potential Fungal UPR Pathway Inhibitors

CharpentierThomas^1^RichommeAnne-Marie Le Ray^1^Bataillé-SimoneauNelly^2^HélesbeuxJean-Jacques^1^SéraphinDenis^1^SimoneauPhilippe^2^GuillemetteThomas^2^RichommePascal^1^ViaultGuillaume^1^1Université Angers, SONAS, SFR QUASAV, 49000 Angers, France; e-mail: guillaume.viault@univ-angers.fr2Université Angers, Institut Agro, INRAE, IRHS, SFR QUASAV, 49070 Beaucouzé, France

**Figure 30:**
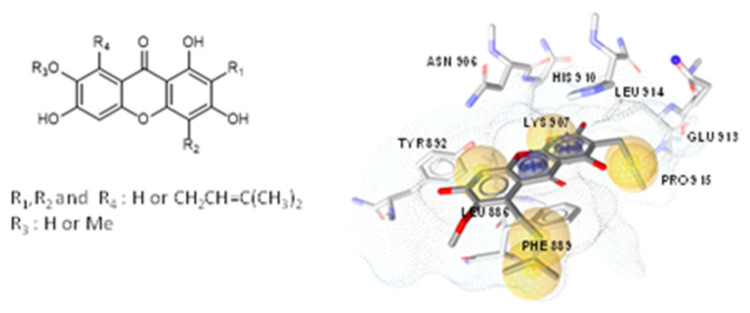
Structure of synthesised xanthones and docking post of α-mangostin in the cytosolic domain of IRE1.

The Unfolded Protein Response (UPR) is essential in the control of Endoplasmic Reticulum (ER) protein folding and maturation for eukaryotes [146]. As far as phytopathogens are concerned, the UPR is also an important pathway associated with antifungal resistance. Indeed, UPR depletion in *Alternaria brassicicola* mutants resulted in a complete loss of virulence as well as a high sensitivity to phytoalexins [147]. Therefore, the UPR pathway appears as a promising target for the development of new antifungal drugs. In fungi, the UPR signal pathway is only mediated by the transmembrane protein IRE13. Testing 76 naturals products (terpenoids, alkaloids and polyphenols) on cell-based screening assay led to the identification of seven potential inhibitors of IRE1, including 4 polyhydroxylated and prenylated xanthones. Antifungal activity of the latter was finally assessed on cabbage leaves infected by A. brassicicola [148]. Results from this assay highlighted that the number and the position of prenyl side chains modulate the inhibitory potential against IRE1 for the corresponding derivatives. To better understand the structure-activity relationships (SAR) a selection of 10 prenylated analogues from the xanthone series have been synthesized. Then, IRE1 inhibition was assessed in a cell-based assay and a molecular docking study was performed (Figure 30). 

### 4.25. Chromanones as a Privileged Scaffold for Multineurotarget Anti-Alzheimer Agents

KeulerTim^1^LemkeCarina^1^Deuther-ConradWinnie^2^Marco-ContellesJosé^3^GütschowMichael^1^1Pharmaceutical Institute, Pharmaceutical & Medicinal Chemistry, University of Bonn, An der Immenburg 4, 53121 Bonn, Germany; e-mail: tkeuler@uni-bonn.de2Helmholtz-Zentrum Dresden-Rossendorf, Institute of Radiopharmaceutical Cancer Research, Department of Neuroradiopharmaceuticals, 04318 Leipzig, Germany3Laboratory of Medicinal Chemistry, IQOG, CSIC, C/Juan de la Cierva 3, 28006 Madrid, Spain

The multifactorial nature of Alzheimer’s disease necessitates the development of agents able to interfere with different relevant targets [149]. Hence, the corresponding design has to combine diverse pharmacophores structurally overlapping or separated by appropriate linkers. For this purpose, a series of 22 tailored chromanones was conceptualized and synthesized. 

The final compounds were prepared from 6-hydroxychroman-4-one, whose phenolic group was converted to an ether moiety, either by a Mitsunobu-Tsunoda protocol [150] or Williamson chemistries [151]. The final set of chromanones was subjected to biological evaluation. Acetyl- and butyrylcholinesterase (AChE and BuChE) activities were monitored with the appropriate thiocholine substrates [152]. Activities of monoamine oxidases A and B (MAO-A, MAO-B) were followed in a peroxidase-coupled assay with tyramine as substrate [153]. Affinity to the s1 and s2 receptors was determined with radioligand displacement assays. 

**Figure 31:**
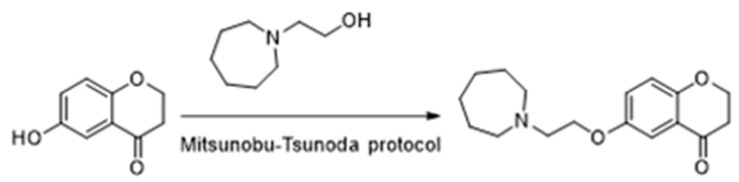

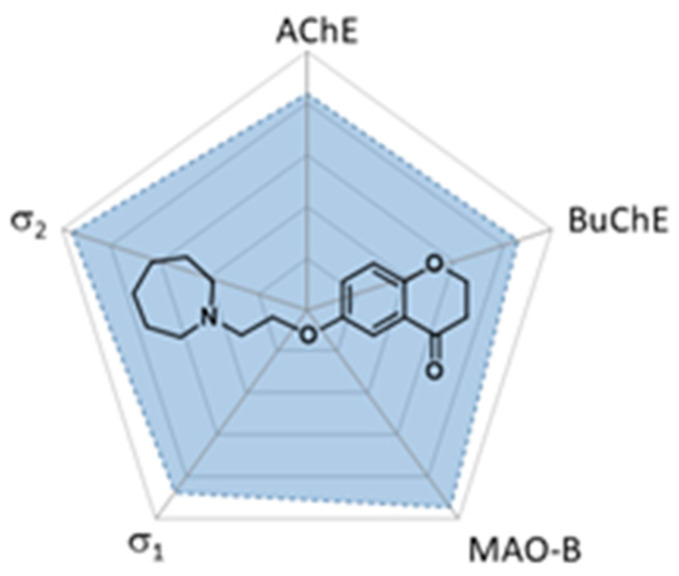
Chromanones as a Privileged Scaffold for Multineurotarget Anti-Alzheimer Agents.

We identified one representative bearing a linker-connected azepane moiety (compound 19) with a balanced pharmacological profile (Figure 31). Compound 19 exhibited inhibitory activities against human AChE and BuChE (IC_50_ < 20 µM) and MAO-B (IC_50_ = 1.1 µM), as well as high affinity to both the s1 and s2 receptor (Ki < 200 nM). Our study provides a framework for the development of further chromanone-based multineurotarget agents. 

### 4.26. Design, Synthesis, and Enzymatic Evaluation of New HDAC 1 and 2 Orto-Aminobenzamides Inhibitors

PavanAline Renata^1^,^2^AlbuquerqueGabriela R.^1^UriasBeatriz S.^1^ProkopczykIgor M.^1^AlvesTânia^1^MeloThais R. F.^1^SantosJean Leandro dos^1^1Department of Drugs and Medicines, São Paulo State University (UNESP), School of Pharmaceutical Sciences, Araraquara 14800-903, Brazil. e-mail: aline.pavan@unesp.br2Institute of Chemistry, São Paulo State University (UNESP), Araraquara 14800-900, Brazil

The epigenetic regulation in the cells is based in a balance among “writers”, “readers” and “eraser” complexes responsible for directing transcription factors that will result in genes expression or suppression [154]. Histone deacetylases are “erasers” enzymes that work through the cleavage of acetyl groups from histone tails in the nucleosome, which restores the charges in this area, increases the interaction between the DNA and the histone tails and end up in gene silencing. A misbalance in the production or in the activity of these enzymes results in important changes in the cells homeostasis leading to a variety of diseases [155]. Nowadays, four HDAC inhibitors were FDA-approved for the treatment of cancer [156]; however, their lack of selectivity among the 18 known HDACs results in undesired effects. Aiming to achieve HDAC 1 and 2 selective inhibitors, this work designed eight new molecules with an orto-aminobenzamide moiety by using docking studies. Appling molecular docking to hint an interaction pattern between the ligands and HDACs 1 and 2 it was observed, in general, that all ligands occupy the active site through the same manner. The 2-aminobenzamide moiety was sited into cavity been able to coordinate with Zn^2+^ as expected. The presence of unsaturation drove the ligands toward exposure to solvent and not realizing interactions with target. In other hand, in absence of rigidity imposed by unsaturation, the ligands were able to reach different regions. Looking the exposed cavity, on HDAC 1 the compounds 5 and 24 were able to realize p-p interaction between substituted phenyl ring and the residue TYR204. Besides, compound 24 makes H-bond interaction with HIS178 and backbone of LEU271. On HDAC 2, compound 24 realize only H-bond interaction with HIS183. In other hand, compound 5 present p-p interaction with residue TYR209. All compounds were successfully synthesized and characterized by ^1^H and ^13^C NMR. In a structure-activity relationship it was possible to identify that the molecules with conformational restriction, such as 4 and 7 presented inhibition values of 45 and 42% against HDAC 1 and 28 and 32% against HDAC 2, which are values up to three times lower in comparison with molecules with flexibility, demonstrating that for HDAC inhibitors the unsaturation or any other strategies to restrict the flexibility result in lower inhibition. In order to demonstrate this fact, compound 4 was reduced by catalytic hydrogenation resulting in compound 5, with a simple bond and no conformational restriction. The inhibition value of compound 5 was 92% against HDAC 1 and 93% against HDAC 2 at 10 µM, which prove the necessity of flexible molecules for HDAC inhibition. 

Acknowledgements: FAPESP: Grants: 2015/19531-1; 2018/19523-7; 2021/10059-9, CNPq: Grant 302689/2020-6]; and CAPES–Finance Code 001. 

### 4.27. Enzyme Cascade Converting Cyclohexanol into ε-Caprolactone Coupled with NADPH Recycling Using Surface Displayed Alcohol Dehydrogenase and Cyclohexanone Monooxygenase on E. coli

TianHaijin^1^FurtmannChristoph^1^LenzFlorian^1^SrinivasamurthyVishnu^2^BornscheuerUwe T.^2^JoseJoachim^1^1Institute of Pharmaceutical and Medicinal Chemistry, University of Münster, D-48149 Münster, Germany. e-mail: haijin.tian@uni-muenster.com2Institute of Biochemistry, University of Greifswald, D-17489 Greifswald, Germany

**Figure 32:**
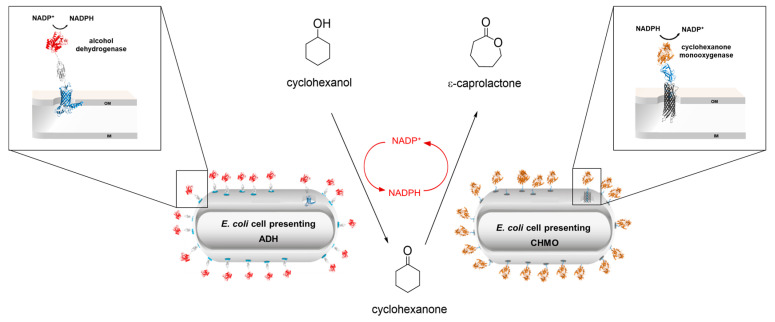
Conversion of cyclohexanol to ε-caprolactone with E. coli cells displaying the ADH and E. coli cells displaying the CHMO. The ADH oxidizes cyclohexanol with the cofactor NADP+, resulting in the products cyclohexanone and NADPH. These are then converted by the CHMO into ε-caprolactone and NADP+.

The application of enzymes as biocatalysts in industrial processes has great potential due to their outstanding stereo-, regio-, and chemoselectivity [157]. Using autodisplay, enzymes can be immobilized on the cell surface of Gram-negative bacteria such as *Escherichia coli* [54,158]. In the present study, the surface display of an alcohol dehydrogenase (ADH) and a cyclohexanone monooxygenase (CHMO) on *E. coli* was investigated (Figure 32). Displaying these enzymes on the surface of *E. coli* resulted in whole cell biocatalysts accessible for substrates without further purification. An apparent maximal reaction velocity *V*_MAX(app)_ for the oxidation of cyclohexanol with the ADH whole cell biocatalysts was determined as 59.9 mU ml^−1^. For the oxidation of cyclohexanone with the CHMO whole cell biocatalysts a *V*_MAX(app)_ of 491 mU ml^−1^ was obtained. A direct conversion of cyclohexanol to ε-caprolactone, which is a known building block for the valuable biodegradable polymer polycaprolactone, was possible by combining the two whole-cell biocatalysts. Gas chromatography was applied to quantify the yield of ε-caprolactone. 1.12 mM ε-caprolactone was produced using ADH and CHMO displaying whole cell biocatalysts in a ratio of 1:5 after 4 h in a cell suspension of OD_578nm_ 10. Furthermore, the reaction cascade as applied provided a self-sufficient regeneration of NADPH for CHMO by the ADH whole cell biocatalyst [159].

### 4.28. Determination and Comparison of Kinetic Parameters for CK2 Enzyme Variants CK2α, CK2α’, CK2α2β2 and CK2α’2β2 in a Universal Buffer

NickelsenAnna^1^FurtmannChristoph^1^LindenblattDirk^2^NiefindKarsten^2^JoseJoachim^1^1Institute of Pharmaceutical and Medicinal Chemistry, PharmaCampus, University of Münster, D-48149 Münster, Germany. e-mail: anna.nickelsen@uni-muenster.de2Institute of Biochemistry, Department of Chemistry, University of Cologne, D-50674 Cologne, Germany

CK2 is a S/T-protein kinase that was investigated as target in different diseases such as cancer and SARS-CoV-19 infection [160]. In humans, there are at least four enzyme variants that are prominent, the free catalytic subunit CK2α, its isoform CK2α’ and the two corresponding holoenzymes consisting of two catalytic subunits binding to a non-catalytic CK2β dimer (CK2α_2_β_2_ and CK2α’_2_β_2_) [161]. The aim of this work was to compare the kinetic parameters such as K_M_ and v_max_ values of the four enzyme variants. Therefore, computer-assisted design of experiments was used for optimizing CK2 activity determination. Based on this and in order to maintain a high level of comparability, a buffer composition universally applicable for all CK2 enzyme variants was elaborated. K_M_ values of the substrate peptide RRRDDDSDDD and the co-substrate ATP were determined with this new buffer composition. Thereby the free catalytic subunits showed higher K_M_ values for the substrate as the corresponding holoenzymes. For the co-substrate ATP, an opposite observation was made. Concerning the isoforms, the substrate showed higher affinity to CK2α and CKα_2_β_2_ than to CK2α’ and CKα’_2_β_2_. In contrast, the co-substrate ATP showed affinity to CK2α’ than to CK2α. However, this difference was not detectable for the holoenzymes. The K_M_ values as determined were compared to the K_M_ values described in the literature, to get an impression on the influence of buffer compositions used in the past. Finally, the new buffer composition and concentrations of substrate and co-substrate adjusted according to the K_M_ values as determined, were analysed on their influence on CK2 inhibition. It turned out that this had no significant impact on the IC_50_ value determination of the known CK2 inhibitors CX-4945 [162] and TBB [163].

### 4.29. Can We Expect Novel Chemical Structures from Highly Studied Fungal Strains? The Case Study of Penicillium Chrysogenum

WatierEmelineLogodinEnoraLescautNatachaRoullierCatherineGentilEmmanuelRuizNicolasGrovelOlivierPouchusY. FrançoisBertrandSamuelNantes Université, Institut des Substances et Organismes de la Mer, ISOMer, UR 2160, F-44000 Nantes, Francee-mail: samuel.bertrand@univ-nantes.Fr

Nowadays, natural products (NP) are still a great source of insparation for drug discovery process. However, research is still largely based on random screening of microbial extracts. This strategy is costly and time-consuming and is one reason of the current decrease and mutation of NP research in pharmaceutical companies. 

As a main strategies, NP chemists rely mostly on the search for new microbial species, to look for novel species. However, with new genomic and metabolomic approaches, new insight within chemical diversity produced by known microorganisms are possible. 

Thus, we initiated an in-depth study of the fungus *Penicillium chrysogenum*, known as a penicillin producer. We use genome annotation using FungiSmach completed by chemical annotation of fungal extract composition after LC-HRMS profiling to show an unprecedented chemical diversity that could be expected from such organisms.

In conclusion, new chemical entities could be expected as a novel sources of chemical structures to be evaluated for drug discovery.

This project is funded by the ANR-18-CE43-0013-01–FREE-NPs project.

### 4.30. Taking Back Control of the Immune System

HaywardDeanneSearceyMarkBeekmanAndrewSchool of Pharmacy, University of East Anglia, Norwich Research Park, Norwich NR4 7TJ, UK; e-mail: d.hayward@uea.ac.uk

Immunotherapy provided a turning point in oncology, transforming treatment efficacy with a more targeted approach than conventional treatments [164]. By blocking the interaction between the immune checkpoint molecule and its ligand, T cells can be activated to eliminate tumour cells. Programmed cell death 1 (PD-1) and programmed cell death ligand 1 (PD-L1) are well described checkpoint proteins that have been successfully targeted with monoclonal antibodies [165,166].

**Figure 33:**
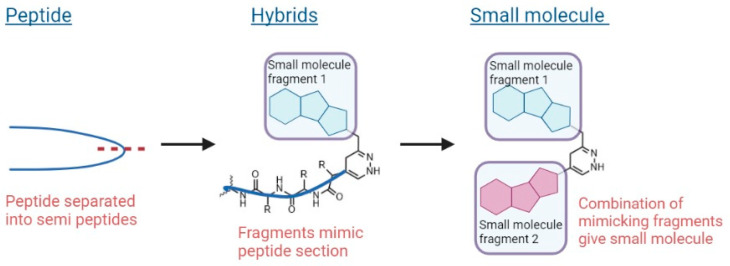
Peptide directed binding.

Small molecules offer an alternative to antibodies to modulate the protein-protein interaction between PD-1 and PD-L1. However, to date, there have been no reports of small molecules with proven binding to either protein. Bristol-Myers Squibb reported cyclic peptides that show tight binding to PD-L1 in the PD-1 binding site. Through peptide directed binding (Figure 33), these cyclic peptides will be used as scaffolds to create small molecule drug leads while maintaining original binding properties [16].

The synthesis of cyclic peptides will be discussed alongside the steps towards peptide directed binding to synthesise novel small molecule inhibitors of PD-1/PD-L1. To mimic the turn of the peptides, click chemistry will be utilized using tetrazines which will be conjugated to the small molecule fragments. Azobenzenes will also be explored to mimic this turn in use with photochemistry.

### 4.31. Discovery, Synthesis and Biological Evaluation of Indolyl-Diketone Derivatives as Cannabinoid Receptor Agonists

PikullikCarolin S.PerriFilomenaMaharadhikaAndhika B.KremersSarah E.RessemannAnastasiiaBoshtaNader M.MüllerChrista E.Pharmaceutical & Medicinal Chemistry, PharmaCenter Bonn, Pharmaceutical Institute, Rheinische Friedrich-Wilhelms-Universität Bonn, An der Immenburg 4, D-53121 Bonn, Germany; e-mail: carolin.pikullik@uni-bonn.de, fperri@uni-bonn.de

**Figure 34:**
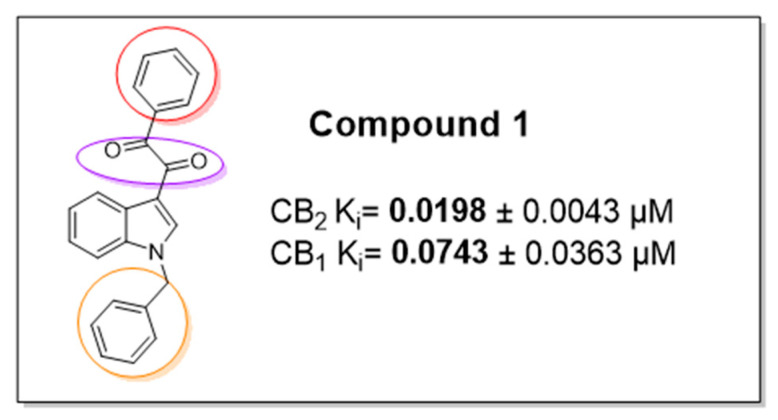
Structure of compound **1**.

Cannabinoid (CB) receptors are subdivided into two subtypes, CB_1_ and CB_2_, both of which belong to the superfamily of G protein-coupled receptors (GPCRs). They are involved in a variety of key physiological processes including cognitive, nervous, metabolic and immune functions. Thus, modulators of both cannabinoid receptor subtypes are of growing interest in drug development for various promising therapeutic approaches (e.g., obesity, nausea, mood and anxiety disorders, (neuro)inflammation including multiple sclerosis and neuropathic pain) [167]. A screening campaign of an indole sub-library of the PharmaCenter Bonn compound library revealed that compound **1** displayed nanomolar affinity for CB_1_ (0.0743 ± 0.0363 µM) and CB_2_ receptors (0.0198 ± 0.0043 µM) determined in radioligand binding studies (Figure 34). CB receptor agonists based on an indole scaffold have previously been described [168]. However, indolyl-diketones such as compound **1** are so far only known as modulators of adenosine receptors [169], to the best of our knowledge, and they therefore represent a new scaffold for CB receptor ligands. Structure-activity relationships around hit compound **1** were explored by synthesizing series of derivatives with modifications of the different substituents as well as the diketone linker. 

CB receptor affinity was lost when the phenylethyldione moiety was removed or exchanged for a smaller polar residue showing that the 1,2-diketone linker plays a crucial role for CB_1_ and CB_2_ receptor affinity as well as agonistic activity (determined in cAMP accumulation and β-arrestin recruitment assays). The novel compounds have provided insights into the structural requirements for this class of CB_1_ and CB_2_ receptor ligands. They will serve as novel lead structures for the future design and development of potent CB receptor agonists.

### 4.32. C6-Modulation and Scaffold Hopping of Theinopyrimidinone Antiplasmodial Hit with Multi-Stage Activity

RomainMustière^1^SébastienHutter^2^VivianaDell’Orco^1^NadiaAmanzougaghene^3^ShahinTajeri^3^CélineDeraeve^4^NadineAzas^2^PierreVerhaeghe^4^PatriceVanelle^1^DominiqueMazier^3^NicolasPrimas^1^1Aix Marseille Université, CNRS, ICR UMR 7273, PCR, Faculté de Pharmacie, Marseille, France; Nicolas.primas@univ-amu.fr2Aix Marseille Université, IHU Méditerranée Infection, UMR VITROME, Marseille, France3Sorbonne Université, CNRS/INSERM, CIMI, Paris, France4Université Paul Sabatier, CNRS UPR 8241, LCC, Toulouse, France

Malaria is a parasitic infection caused by *Plasmodium* that affected 229 million people and killed about 409 000 in 2019, according to the WHO [116]. The discovery of new antimalarial compounds is necessary to tackle the spread of artemisinin-resistant *P. falciparum* strains [170]. In this context, we identified M1, a lead compound belonging to the 2-aminothieno [3,2-d]pyrimidinones series showing in vitro multi-stage antiplasmodia activity associated with low cytotoxicity [171,172]. Moreover, M1 is active against quiescent artemisinin-resistant *P. falciparum* strain. Unfortunately, M1 is quickly metabolized by mouse liver microsome into inactive derivatives (t½ = 11 min), leading to activity loss in vivo in a mouse model.

**Figure 35:**
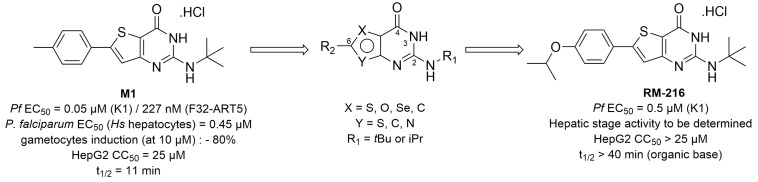
Structure of Hit M1 and chemical modulation at position 6 of the scaffold.

To improve M1 microsomal stability and to complete the structure-activity relationship (SAR) studies, position 6 of the thieno [3,2-d]pyrimidine scaffold was modulated and a scaffold hopping of the five-membered ring of the thienopyrimidine core was also investigated (Figure 35). All compounds were evaluated in vitro on the erythrocytic stage of *P. falciparum*. Best compounds were further assessed on the hepatic stage of P. berghei and their in vitro metabolic stability was determined. Pharmacomodulations allowed us to discover new molecules with improved metabolic stability while limiting the loss of activity. Synthetic routes and biological results will be presented in the communication.

### 4.33. Improving the Druglikeness of a Nitroimidazopyridine Antileishmanial hit

Paoli-LombardoRomain^1^PrimasNicolas^1^Bourgeade-DelmasSandra^2^HutterSébastien^3^Sournia-SaquetAlix^4^Castera-DucrosCaroline^1^CorvaisierSophie^5^SinceMarc^5^Malzert-FréonAurélie^5^VerhaeghePierre^4^AzasNadine^3^RathelotPascal^1^VanellePatrice^1^1Aix Marseille Univ, CNRS, ICR UMR 7273, PCR, 13385 Marseille, France. e-mail: romain.paoli-lombardo@etu.univ-amu.fr2Université Paul Sabatier, UMR 152 PharmaDev, 31062 Toulouse, France3Aix Marseille Univ, IHU Méditerranée Infection, UMR VITROME, 13005 Marseille, France4Université Paul Sabatier, CNRS UPR 8241, LCC, 31077 Toulouse, France5Normandie Université, UNICAEN, CERMN, 14000 Caen, France

**Figure 36:**
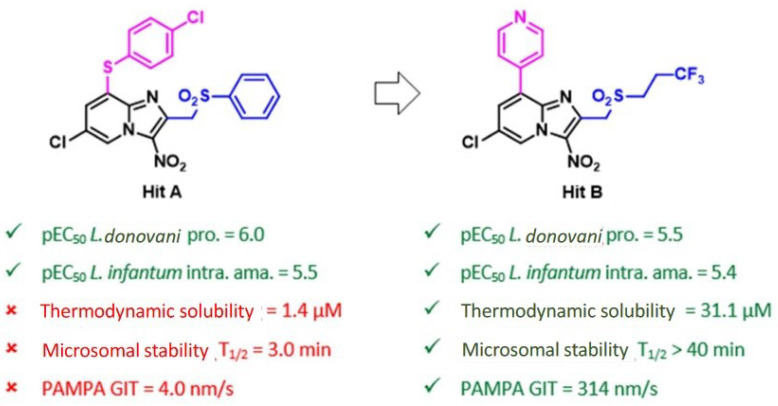
Improvement of in vitro physicochemical and pharmacokinetic properties of antileishmanial hit compound in 3-nitroimidazo [1,2-a]pyridine series.

Life-threatening visceral leishmaniasis (VL) is a vector-borne parasitic disease caused by *Leishmania donovani* and *L. infantum*. With an estimated 50,000–90,000 new cases per year, VL is fatal within two years if left untreated and causes more than 30,000 deaths annually [173]. Moreover, currently available treatments have significant limitations, making the discovery of new efficient, safe and inexpensive antileishmanial drugs a priority. In this context, our team identified an antileishmanial hit compound (Hit A) in 3-nitroimidazo [1,2-*a*]pyridine series bearing a phenylsulfonylmethyl group at position 2 and a 4-chlorophenylthioether moiety at position 8 [174]. Unfortunately, this hit compound showed limited aqueous solubility, a poor mouse liver microsomal stability and a weak gastrointestinal permeability. To improve these parameters, we explored the structure-activity relationship of the substituents at the positions 2 and 8 of the imidazo [1,2-*a*]pyridine ring, leading to the discovery of a new antileishmanial hit (Hit B) with greatly improved solubility, stability and gastrointestinal permeability, bearing a *gem*-trifluoropropylsulfonylmethyl group at position 2 and a pyridin-4-yl moiety at position 8 (Figure 36). The synthesis and structure-activity relationship data will be presented in the communication.

### 4.34. Novel (Semi-)Synthetic Phloretin-Based Inhibitors of AKR1C3 Overexpressed (Castration-Resistant) Prostate Cancer

GhidiniAndrea^1^MollerGabriele^2^PeraltaAntonio Cala^1^ViaultGuillaume^1^SchusterDaniela^3^TemmlVeronika^3^SéraphinDenis^1^DyarKenneth^2^HelesbeuxJean-Jacques^1^1Université Angers, SONAS, SFR QUASAV, F-49000 Angers, France; andrea.ghidini@univ-angers.fr2Research Unit Molecular Endocrinology and Metabolism, Helmholtz Zentrum München, 85764 Neuherberg, Germany3Department of Pharmaceutical and Medicinal Chemistry, Paracelsus Medical University, 5020 Salzburg, Austria

Prostate cancer is nowadays the second most frequent malignancy in men, and the second by death rate, with more than 260,000 new cases expected for 2022 only in the USA [175]. Current therapies with second-generation antiandrogens, such as enzalutamide, provided a great enhancement in the therapeutic arsenal, but these new drugs are already facing the issue of the resistance developed by tumors [176]. 

Various studies already made explicit the important role of the enzyme Aldo-Keto reductase 1C3 (AKR1C3) in the development of the resistance. Among the natural compounds investigated, a 3′-benzylated dihydrochalcone (BnDHC) MF-15 showed attractive antineoplastic activity in a cell model of enzalutamide-resistant prostate cancer [177].

**Figure 37:**
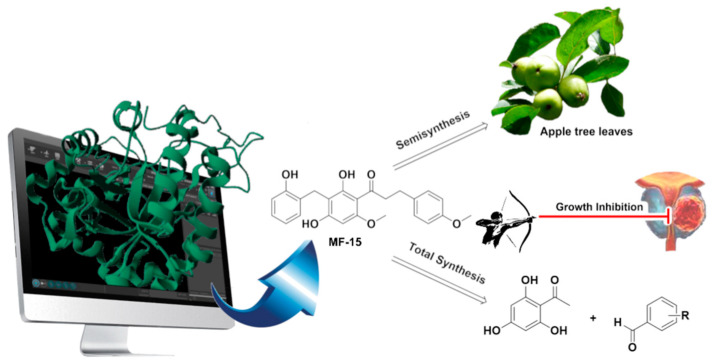
General strategy to develop new MF-15 analogues targeting AKR1C3 and limiting tumor growth.

This project aims to develop new MF-15 analogues targeting AKR1C3 with an improved pharmacological profile. A first set of semisynthetic compounds was obtained starting from the natural dihydrochalcones phloretin and 3-hydroxyphloretin, initially extracted as glycosylated forms respectively from the leaves of *Malus domestica* and *M. sieboldii* (Figure 37). A second set of novel BnDHC was obtained by a total synthesis approach. The activity of new BnDHC has been tested in a preliminary inhibition assay at 10 µM against AKR1C3.

### 4.35. Novel Pleiotropic Compounds Inhibiting 5-HT4 Receptors with Antioxidant Properties for the Treatment of Alzheimer’s Disease

LanthierCaroline^1^LecouteyCédric^1^CiambellaMonica^1^DavisAudrey^1^CurelThomas^2^PayanHugo^2^VignolThomas^1^SinceMarc^1^ClaeysenSylvie^2^DallemagnePatrick^1^RochaisChristophe^1^1Normandie Université, UNICAEN, Centre d’Etudes et de Recherche sur le Médicament de Normandie, 14000 Caen, France;cedric.lecoutey@unicaen.fr2Institut de Génomique Fonctionnelle, Université Montpellier, CNRS, INSERM, 34000 Montpellier, France

Alzheimer’s disease (AD), the main form of dementia, affects more than 50 million people worldwide [178]. The formation of amyloid plaques is one of highlighted molecular causes. On the other hand, researchers have correlated these aggregations with oxidative stress by overproduction of reactive oxygen species (ROS) which leads to the neuronal death [179]. 

The multifactorial nature of AD permits the emergence of a new pharmacological approach: the Multi-Target-Directed Ligands (MTDL) strategy [101]. It consists in developing single molecules able to act on several targets. Then, some compounds with antioxidant properties and able to prevent the formation of amyloid plaques could be promising. For this last target, we focus on the 5-HT4 receptor (5-HT4R) whose activation with partial agonists promotes the non-amyloidogenic cleavage and so decrease the charge of amyloid. 

**Figure 38:**
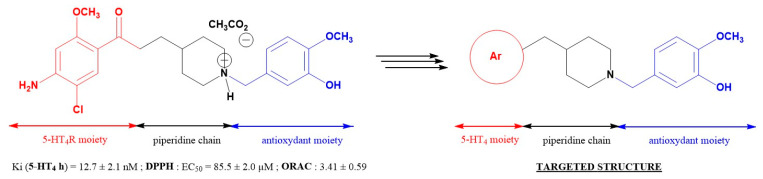
Structural modifications of the hit towards new MTDL.

Some preliminary works highlighted first compounds with this dual activity [180]. From a well-known 5-HT4R scaffold (aminochlorobenzophenone), we have connected different antioxidant scaffold. These two moieties are linked with a piperidine chain, essential for the 5-HT4R activity (Figure 38). Among these MTDL, the isovanilline group appears to be the best compromise for the dual activity. 

In this present work, we perform the reverse work: we have kept the isovanilline moiety for the antioxidant part and we have connected different 5-HT4 scaffolds or commercial benzoic acids with the aim to obtain MTDL. First description of these molecules together with in vitro evaluation will be presented in this communication.

### 4.36. Bisindolylmaleimide Based Heterocyclic Compounds Are Involved in Fluconazole Susceptibility Restoration towards Candida albicans Strains

Ourliac-GarnierIsabelle^1^CooneyLouise^2^AlbassierMarjorie^1^LogéCédric^1^PapePatrice Le^1^MarchandPascal^1^McCarthyFlorence O.^2^1Nantes Université, Cibles et médicaments des Infections et de l’Immunité, IICiMed, UR 1155, F-44000 Nantes, France; isabelle.ourliac@univ-nantes.fr2School of Chemistry, Analytical and Biological Chemistry Research Facility, University College Cork, T12 K8AF Cork, Ireland

In models of experimental infection, strains deleted for elements of MAPK-mediated signal transduction pathways exhibit a reduction or loss of virulence [181]. In *Candida albicans*, protein kinase C (CaPkc1), one of the key proteins involved in MAPK pathways, is described as a regulator of cell wall integrity during growth, morphogenesis and response to cell wall stress [182]. In addition, La Fayette et al. established a new role for PKC signaling in drug tolerance mechanism [183]. Given the limited number of antifungals used in clinics [184] and the emergence of drug resistance [185], there is an urgent need to identify alternative targets in order to speed up the development of new generation of antifungals either more effective or able to restore susceptibility to classical antifungal drugs [186]. In this context, targeting PKC-mediated signal transduction pathway represents a new attractive strategy for antifungal therapy [187].

**Figure 39:**
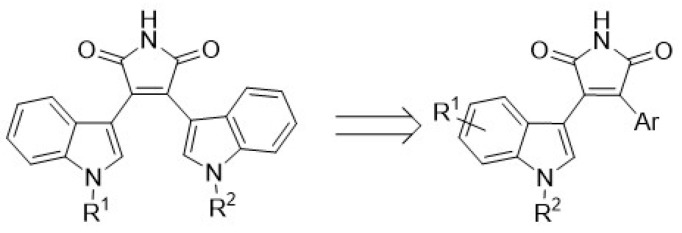
From BIM derivatives to BFIM and NIM analogues.

Members of the chemical class of bisindolylmaleimides (BIM) are well-known potent human protein kinase (PKC) inhibitors [188]. To design new molecules, specifically targeting CaPKC, BIM, BFIM (benzofuranyl) and NIM (naphthyl) derivatives were synthesized (Figure 39). 

To investigate the biological interest of these new molecules, MICs have been determined following an EUCAST modified procedure, on a collection of *Candida albicans* strains selected for their susceptibility or resistance to fluconazole. The potential restoration of fluconazole susceptibility has been investigated in vitro on resistant strains and also in an alternative in vivo model, *Galleria mellonella*. CaPKC as a target of these compounds now needs to be confirmed. 

Acknowledgments: PHC Ulysses Fundings—Application 45387ZJ.

### 4.37. Structure-Inhibition Relationships with Regard to the S2 Site of SARS-CoV-2 Mpro

BreidenbachJulianVogetRabeaVuLan PhuongMüllerChrista E.GütschowMichaelDepartment of Pharmaceutical and Medicinal Chemistry, University of Bonn, Bonn, Germany, An der Immenburg 4. jbreiden@uni-bonn.de

**Figure 40:**
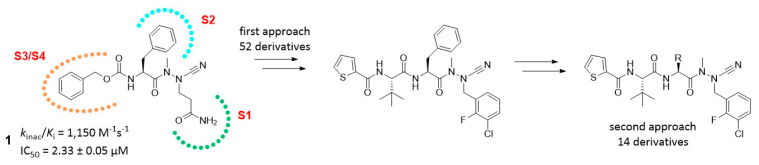
Optimization procedure from the irreversible SARS-CoV-2 Mpro inhibitor 1 to potent, new derivatives.

The continuously occurring variants of SARS-CoV-2 escape the tailored vaccines and entail an unpredictable duration of the COVID-19 pandemic. For this reason, an appropriate treatment of affected patients suitable for current and forthcoming virus variants is indispensable. The main protease (Mpro) constitutes an eligible target; its inhibitors have already shown efficacy against former and present variants of SARS-CoV-2 [189]. For the design of further potent inhibitors, a more detailed insight into the structure-activity relationships on the respective subsites (S1–S4) is inevitable (Figure 40).

Our previous study identified the peptidomimetic azanitrile inhibitor 1 as an irreversible Mpro inhibitor with moderate activity [190]. In a first attempt, we optimized the P1 and P3/P4 position with the aid of 52 azanitrile derivatives and discovered a 3-chloro-2-fluorophenyl moiety at the P1 and an (S)-2-(thiophene-2-carboxamido)-tert-leucine at the P3/P4 position as the most advantageous fragments. The second approach led to generation of further 14 inhibitors, which stand out due to their chemical diversity in the P2 position. On this account, it was possible to gain a deeper insight into the structure-activity relationships with respect to the S2 site of SARS-CoV-2 Mpro. This new subseries of azanitriles encompasses inactive to highly potent representatives. Thus, our biochemical evaluation data point towards the potential and the limitations of the P2 diversification and enable new perspectives in Mpro drug design.

### 4.38. Peptides and Small Molecules Stabilise DNA Four-Way Junctions

SearceyMarkBeekmanAndrewIvensEleanorSchool of Pharmacy, University of East Anglia, Norwich NR4 7UL, UK; e.ivens@uea.ac.uk

Recently, the targeting of higher order DNA structures has been explored as a new approach for the development of more selective therapies, when compared to double-stranded DNA (dsDNA). This has been primarily focussed on the G-quadraplex, with a large number of compounds developed against this target [191]. 

The lesser known and simpler structure of the four-way junction (4WJ) has subsequently become a subject of interest. The 4WJ, also known as the Holliday junction, consists of four strands of DNA joining together during the cross-over of two dsDNA molecules. This is a common intermediate formed during the process of homologous recombination, involved in DNA repair [192]. 

In order to prevent DNA repair in cancer and bacterial cells, studies have focussed on the development of compounds that trap 4WJs, preventing their resolution. This has resulted in the discovery of a potent hexapeptide binder, WRWYCR, which is found to form a dimer within the 4WJ [193]. WRWYCR has been shown to have antibacterial effects against multiple types of bacteria [194]. 

**Figure 41:**
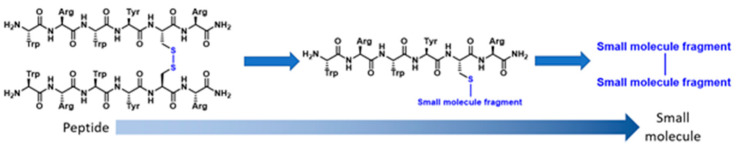
The use of Peptide-Directed Ligand Design for the development of novel small molecule 4WJ binders, from the known dimeric peptide 4WJ binder WRWYCR.

The plan for this PhD will be to move away from peptides to more drug-like compounds. This will involve using the WRWYCR monomer and attaching various chemical groups to see if 4WJ activity is maintained. It is hoped that active chemical groups can be combined or dimerised to form novel small molecule 4WJ inhibitors (Figure 41).

### 4.39. Anti-Inflammatory Potential of Chalcones in the Vitamin E Series

AlsabilKhaled^1^PermannStephan^2^BrunnerElena^3^ViaultGuillaume^1^SchusterDaniela^4^TemmlVeronika^4^Werzd Oliver^3^SeraphinDenis^1^KoeberleAndreas^2^HelesbeuxJean-Jacques^1^1Université Angers, SONAS, F-49000 Angers France2Michael Popp Institute/Center for Molecular Biosciences Innsbruck, University of Innsbruck, 6020 Innsbruck, Austria3Department of Pharm/Med Chem, Institute of Pharmacy, Friedrich Schiller University Jena, 07743 Jena, Germany4Department of Pharmaceutical and Medicinal Chemistry, Paracelsus Medical University, 5020 Salzburg, Austria; khaled.alsabi@univ-angers.fr

Inflammation is a normal body’s response to a harmful stimuli, that can be a pathogen, a physical injury, a chemical, etc. In the first and acute stage of this complex phenomenon, leukocytes infiltrate the damaged region and produce potent pro-inflammatory mediators to favor the removal of the stimuli and the repair of the tissues. Chronic inflammation may occur as a result of a prolonged, dysregulated and maladaptive response, that can thus be a component of diverse pathologies [195]. Leukocytes contain various enzymes such as 5-lipoxygenase (5-LOX) or microsomal prostaglandin E2 synthase type 1 (mPGES-1) that participate to the biosynthesis of pro-inflammatory mediators and that count amongst the validated biological targets to circumvent such chronic process [196].

**Figure 42:**
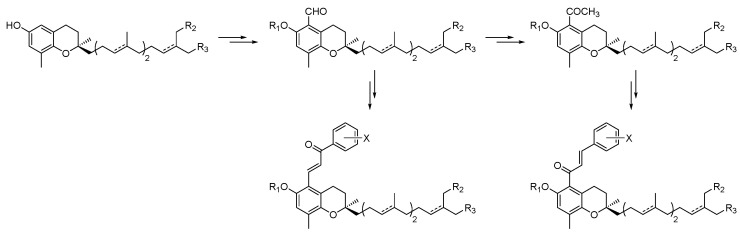
Semisynthetic strategies to access chalcones in the vitamin E series.

Over the past decade, our group has identified several oxidized vitamin E analogues inhibiting these two enzymes. We previously reported an efficient formylation strategy in that series [197]. The corresponding aldehydes have been condensed with various acetophenones to access a first set of chalcone hybrids (Figure 42). These formylated chromanols have also been involved in a two-step strategy to prepare the 5-acetylated derivatives that were eventually used to prepare a second series of chalcone-chromanol analogues. Their inhibitory potential against 5-LOX and mPGES-1 is currently under evaluation. Some preliminary results will be reported. 

Authors would like to thank ANR, DFG and FWF for their financial support of DIVE project.

### 4.40. α-Amplexichromanol as a Highly Potent 5-LOX Inhibitor

AlsabilKhaled^1^NeukirchKonstantin^2^,^3^VilleAlexia^1^ViaultGuillaume^1^SchusterDaniela^4^TemmlVeronika^4^RossiAntonietta^5^RoviezzoFiorentina^5^WerzOliver^3^SeraphinDenis^1^KoeberleAndreas^2^HelesbeuxJean-Jacques^1^1Université Angers, SONAS, F-49000 Angers France; jj.helesbeux@univ-angers.fr2Michael Popp Institute/Center for Molecular Biosciences Innsbruck, University of Innsbruck, 6020 Innsbruck, Austria3Department of Pharm/Med Chem, Institute of Pharmacy, Friedrich Schiller University Jena, 07743 Jena, Germany4Department of Pharmaceutical and Medicinal Chemistry, Paracelsus Medical University, 5020 Salzburg, Austria5Department of Pharmacy, School of Medicine and Surgery, University of Naples Federico II, 80131 Naples, Italy

**Figure 43:**
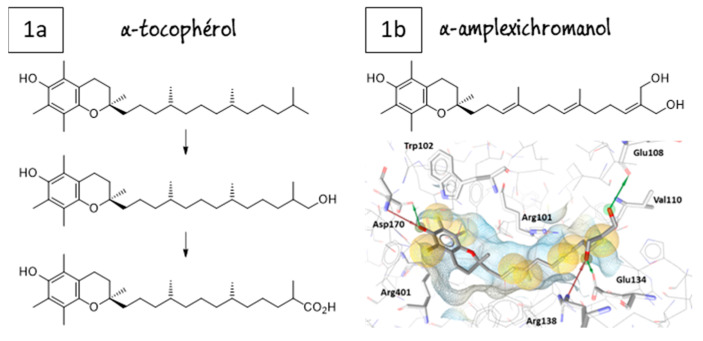
(**1a**) From α-tocopherol to the corresponding ω-alcohol and ω-carboxylic acid. (**1b**) α-amplexichromanol (structure and pose/interactions within the allosteric site of 5-LOX).

Deficiency of vitamin E causes a dysfunctional immune response, degenerative diseases and potentially atherosclerosis [198]. The discovery that the vital antioxidant vitamin E mediates immune functions through endogenous long-chain metabolites (LCMs) recently revived research on this field [199]. LCMs are produced from α-tocopherol and other vitamin E forms by hepatic ω-oxidation, yielding ω-alcohols and then ω-carboxylic acids (Figure 43). 

In the frame of this project, a large library of vitamin E analogues, mainly in the tocotrienol series, has been semi-synthesized (Figure 43). Their 5-lipoxygenase inhibitory potential has been first evaluated in cell-free and cell-based assays. This screening allowed the identification of α-amplexichromanol as a highly potent inhibitor. Hence its pharmacological profile has been further characterized. This study highlighted the selectivity among various targets involved in the metabolism of arachidonic acid, an improved metabolic stability and the in vivo efficacy in a mouse model of asthma of α-amplexichromanol when compared to α-tocopherol and its corresponding oxidized metabolites [200].

Authors would like to thank ANR, DFG and FWF for their financial support of DIVE project.

### 4.41. A Comparison of Chiral Diastereomeric Versus Kinetic Enzymatic Resolution for Enantioseparation of Microtubule Depolymerising Beta-Lactams

McLoughlinEavan C.^1^BarroetaPatricia Hannon^2^ZistererDaniela M.^2^O’BoyleNiamh M.^1^1School of Pharmacy and Pharmaceutical Sciences, Panoz Institute and Trinity Biomedical Sciences Institute, Trinity College Dublin, Dublin, Ireland; mclougea@tcd.ie2School of Biochemistry and Immunology, Trinity Biomedical Sciences Institute, Trinity College Dublin, Dublin, Ireland

**Figure 44:**
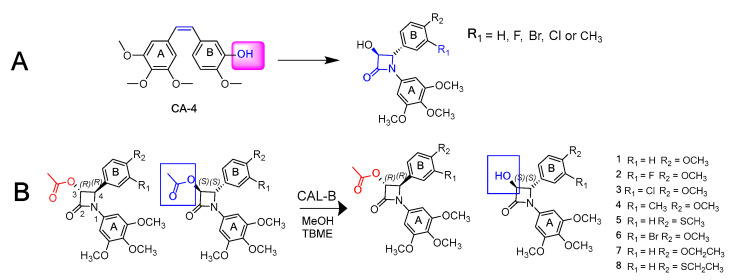
(**A**) Structural modifications to prevent cis/trans isomerisation of CA-4 and increase anti-proliferative activity in CA-4 resistant HT-29 cells; (**B**) CAL-B mediated methanolysis of 3-acetoxy to *3S,4S* 3-hydroxyl enantiomers.

A panel of 3-hydroxyl B ring meta hydroxyl substituted β-lactam racemates of combretastatin A-4 (CA-4), known as ‘the combretazets’ have demonstrated microtubule depolymerising activity in both breast cancer and CA-4 resistant colorectal cancer cells (Figure 44A) [201,202]. Chiral diastereomeric resolution has successfully resulted in enantioseparation of + and–3-hydroxyl β-lactams as the N-(Boc)-l-proline derivatised *3S,4S* and *3R,4R* diastereomers using liquid chromatography (LC) [203]. While diastereomeric resolution resulted in enantioseparation of the *3S,4S* enantiomer in >90% enantiomeric excess (*ee*), co-elution of diastereomers resulted in lower *ee* of 50-80% for the *3R,4R* diastereomer. LC chiral resolutions were also labour and time intensive, requiring large solvent volumes while yielding only minimal quantities of enantiopure β-lactams. The biocatalytic approach involving kinetic enzymatic resolution (KR) for the preparation of enantiomers aimed to improve accessibility, in a more sustainable manner while also increasing enantiomer isolated yields. Industrial processes commonly employ biocatalysts for highly selective and greener economical outputs (Pellis, A., et al. *New Biotechnology,* 2018, *40*, 154-169). Lipase enzymes (EC 3.1.1.3) are valuable biocatalytic enzymes due to their broad substrate specificity and commercial availability. Recombinant Lipase B *Candida antarctica* (CAL-B), immobilised on Immunobead 150, catalyses KR of 3-acetoxy β-lactams (Figure 44B) toward the enantioenriched 3-hydroxyl *3S,4S* β-lactam enantiomer via methanolysis in 70–80% *ee*, comparable to *ee* achieved via diastereomeric chiral resolution. Superior *ee* values of 95% were determined for the unreacted 3-acetoxy *3R,4R* enantiomer. KR of combretazet β-lactams is a more accessible and greener enantioseparation strategy versus diastereomeric resolution, eliminating the requirement for significant purification expertise while also yielding quantities of enantiopure β-lactams necessary for further pre-clinical in vivo biological evaluation.

Acknowledgements: This research was supported by the Royal Society of Chemistry Research Fund (project number R19-5845), Wellcome Trust (Grant Ref [20]4814/Z/16/Z) and Trinity College Dublin’s Provost’s PhD Project Award.

## 5. Conclusions

The GP_2_A 30th annual conference was successfully held in Dublin, Ireland from 24 to 26 August 2022. We are grateful to our sponsors for their support for the event: ThermoFisher, Fáilte Ireland, Collaborative Drug Discovery (CDD Vault), CEM, Biopharma Group, LabCup, Enanime, Biotage, Almac Group, Key Organics, Labplan, Mason Technology, GPE Scientific Ltd., Pharmaceuticals, Lilly, Lennox, Fluorochem, European Journal of Medicinal Chemistry and RSC Medicinal Chemistry. We also acknowledge the staff and students of the School of Pharmacy and Pharmaceutical Sciences and Trinity Central Events (Liane Donnelly and Fiona O’Sullivan) for their help.

The 31st edition of the GP_2_A conference will be held in Marseille from 23 to 25 August 2023, hosted by Aix-Marseille University. We welcome you to the conference!

## Data Availability

Not applicable.
